# Transcriptional regulations of pollen tube reception are associated with the fertility of the ginger species *Zingiber zerumbet and Zingiber corallinum*

**DOI:** 10.3389/fpls.2023.1099250

**Published:** 2023-05-10

**Authors:** Shan Liang, Ming-li Hu, Hao-chuan Lin, Han-jun He, Xi-ping Ning, Pei-pei Peng, Guo-hui Lu, Shu-lan Sun, Xiao-jing Wang, Ying-qiang Wang, Hong Wu

**Affiliations:** ^1^Guangdong Provincial Key Laboratory of Biotechnology for Plant Development, School of Life Sciences, South China Normal University, Guangzhou, China; ^2^State Key Laboratory for Conservation and Utilization of Subtropical Agro-bioresources, College of Life Sciences, South China Agricultural University, Guangzhou, China; ^3^School of Pharmacy, Xianning Medical College, Hubei University of Science and Technology, Xianning, China

**Keywords:** *Zingiber*, double fertilization, FERONIA, ANXUR, pollen tube rupture, sexual reproduction, RNA-Seq, transcriptional regulation

## Abstract

*Zingiber zerumbet* and *Zingiber corallinum* are economically valuable species in the genus *Zingiber*. While *Z. corallinum* is sexually active, *Z. zerumbet* adopts clonal propagation, although it has the potential for sexual reproduction. It is unclear so far at which step during the sexual reproduction of *Z. zerumbet* inhibition occurs, and what are the regulatory mechanisms underlying this inhibition. Here, by comparing with the fertile species *Z. corallinum* using microscopy-based methods, we show that rare differences were observed in *Z. zerumbet* up to the point when the pollen tubes invaded the ovules. However, a significantly higher percentage of ovules still contained intact pollen tubes 24 h after pollination, suggesting pollen tube rupture was impaired in this species. Further RNA-seq analysis generated accordant results, showing that the transcription of *ANX* and *FER*, as well as genes for the partners in the same complexes (e.g., BUPS and LRE, respectively), and those putative peptide signals (e.g., RALF34), were timely activated in *Z. corallinum*, which ensured the pollen tubes being able to grow, reorient to ovules, and receipt by embryo sacs. In *Z. zerumbet*, genes for these complexes were cooperatively suppressed, which would result in the maintenance of PT integrity due to the disruption of RALF34-ANX/BUPS signaling in PT and the failure of PT reception by an active synergid due to the insufficiency of the synergid-harbored FER/LRE complex. Taking the results from the cytological and RNA-seq studies together, a model is proposed to illustrate the possible regulation mechanisms in *Z. zerumbet* and *Z. corallinum*, in which the regulations for pollen tube rupture and reception are proposed as the barrier for sexual reproduction in *Z. zerumbet*.

## Introduction

Many species of flowering plants use crossing to produce hybrid offspring in nature, which is borrowed to generate novel cultivars with advantages over desired agronomic traits or tolerance to abiotic and biotic stress. However, due to the intra- or inter-species fertilization barriers, this is impractical for some agriculturally or pharmaceutically valuable species. As the key step to successful sexual reproduction, double fertilization in angiosperms relies on a series of events that happen sequentially before and after pollination (Johnson et al., 2019). Improper control at any of these steps will result in sexual abortion (Christensen et al., 1998; Capron et al., 2008; Miyazaki et al., 2009; Okuda et al., 2009). Using model plants, several pre-fertilization reproductive barriers are revealed (Kessler and Grossniklaus, 2011; Li et al., 2016; Johnson et al., 2019).

A pollen tube (PT) growing within the transmitting tract can be controlled by specialized tissues, which is a site for regulation of self-incompatibility. Glycoproteins, special amino acids, and adhesins are identified for this regulation, and a lack of the functional forms of these regulators could result in abnormal PT growth (Cheung et al., 1995; Mollet et al., 2000; Park et al., 2000; Palanivelu et al., 2003; Michard et al., 2011). Importantly, PT growth is regulated by a signal cascade, “RALFs-ANX/BUPS-RopGEFs.” The ANX/BUPS complex is located on the membrane of PTs, which is comprised of the receptor-like protein kinase ANXUR1 (ANX1) or ANX2 and its interaction partner Buddha’s Paper Seal1 (BUPS1) or BUPS2. This complex can be recognized by RALF4 or RALF19, two members of the rapid alkalinization factors (RALFs) family in *Arabidopsis*, and in turn trigger downstream ROP (Rho of Plant) Guanine Nucleotide Exchange Factors (RopGEFs)-mediated signaling, resulting in adjustment of PT behaviors to sustain PT growth and integrity (Miyazaki et al., 2009; Ge et al., 2017; Mecchia et al., 2017; Zhu et al., 2018; Xiao et al., 2019; Zhou and Dresselhaus, 2019). Interaction between RALF19 and the ANX/BUPS complex is stabilized by the participation of the LRX8 protein (also known as *PEX1*) (Mecchia et al., 2017; Fabrice et al., 2018), which also contributes to the integrity of PTs during growth. The LORELEI-like GPI-anchored proteins 2 (LLG2) and LLG3 act as chaperones for the secretion of ANX/BUPS to the apical membrane of PT (Feng et al., 2019; Ge et al., 2019b).

Reorientation of PT growth is the next critical step for reproductive control, which ensures that PTs go through the funiculus towards the ovules after exiting the transmitting tract. Funiculus guidance and micropylar guidance are the two sub-steps, which are distinctive in the PT phenotypes and can be regulated by different subsets of genes (Kanaoka and Higashiyama, 2015). So far, only a small number of genes have been verified to be responsible for funiculus guidance, including *MPK3* and *MPK6* for mitogen-activated protein kinase (MPK) proteins, *MAA3* for helicase, and *DHQS* for 3-dehydroquinate synthase (Shimizu and Okada, 2000; Guan et al., 2014; Wang et al., 2023). Many proteins are found to be associated with micropylar guidance, and their loss of function mostly results in reproductive abortion. For example, the EGG APPARATUS 1 (EA1) from maize and the LUREs from *Arabidopsis thaliana* or *Torenia fournieri* serve as guidance cues for this steering. They have diverged extensively across species and can confer an interspecies barrier to hybridization (Márton et al., 2005; Okuda et al., 2009; Márton et al., 2012; Takeuchi and Higashiyama, 2012). Consistently, receptors for these guidance cues are also related to micropylar guidance (Okuda et al., 2009; Takeuchi and Higashiyama, 2012; Cheung and Wu, 2016; Takeuchi and Higashiyama, 2016; Wang et al., 2016). An example can be found in the homodimer or heterodimer complex of PRK proteins (e.g., PRK6 and PRK3) in *Arabidopsis*, which can recognize and bind AtALURE1.2 and consequently activate the downstream signaling (Malho and Trewavas, 1996; Yu et al., 2018). AtLURE1.2 also can be perceived by the MALE DISCOVERER1 (MDIS1), a PT-localized protein that interacts with MDIS1-interacting receptor-like kinase1 (MIK1) or MIK2 to trigger signaling in a phosphorylation-dependent manner (Wang et al., 2016). In addition to LUREs, XIUQIUs are another group of maternally derived peptides that can also be perceived by PRK6 and are involved in PT guidance in *Arabidopsis*. These peptides are evolutionarily conservative, regardless of species, and are considered ancient guidance cues (Zhong et al., 2019).

Upon arrival at the female gametophyte, the growth of PTs becomes slow and ultimately stops at the receptive synergid (Johnson et al., 2019). Reception of PT by female gametophytes serves as another checkpoint, which is often in relation to the overgrowth of PTs within the embryo sac in interspecies crosses (Williams et al., 1982; Escobar-Restrepo et al., 2007). Once the PT invades the embryonic sac, the interaction between the PT and synergid cells must be fine-tuned to ensure the reception of PT by the receptive synergid. A pair of plasma membrane-localized receptor-like kinase complexes ensure the success of PT reception (Miyazaki et al., 2009; Kessler and Grossniklaus, 2011; Ge et al., 2017). One side of this interaction is the PT-associated ANX/BUPS complex; another side is the synergid-associated FER/LRE complex, which contains FERONIA (FER) and its chaperone LORELEI (LRE). FER is a sister protein of ANX1 and ANX2 and is a crucial factor in female fertility by mediating the female control of male gamete delivery during fertilization (Huck et al., 2003; Boisson-Dernier et al., 2009). Like ANX1 can interact with LLG2 or LLG3 in the PT (Feng et al., 2019), the FER protein interacts with LRE directly, by which FER is transported to the plasma membranes of synergids with the help of LRE (Li et al., 2015; Li et al., 2016). Both the ANX/BUPS complex and the FER/LRE complex can perceive the signal peptides of the RALF family. RALF34 is derived from ovules and acts as the competitor of RALF4/19 for the binding site on the ANX/BUPS complex to promote the rupture of PT during its interaction with synergid cells (Kessler and Grossniklaus, 2011). At the same time, RALF34 is also bound by the synergid-associated FER/LRE complex, which cooperatively promotes the interaction between the PT and the receptive synergid.

Although the mechanisms of sexual reproduction have been extensively discovered in model plants, little is known in diverse non-model species due to the lack of spontaneous mutants and efficient techniques for in-depth studies. In the genus *Zingiber* of the pan-tropical family Zingiberaceae, a variety of reproductive strategies are adopted by different species, including sexuality, clonality, or a combination (Kavitha et al., 2010; Thomas et al., 2016). This makes this genus a good model for exploring the genetic controls on the selection of reproduction manners. Clonality is usually the main strategy in those economically important ginger species, such as *Zingiber officinale*, *Zingiber zerumbet*, and *Zingiber corallinum*, which are widely cultivated in tropical and subtropical regions and used for ornamental and medicinal purposes (Koga et al., 2016; Sharifi-Rad et al., 2017). This propagation strategy is beneficial for the conservation of prominent traits, but it is also a significant barrier to novel cultivar breeding. In this study, we focused on *Z. zerumbet* (ZZ) and *Z. corallinum* (ZC), the two closest relatives of this genus (Fang, 1982; Wu and Larsen, 2000; Theerakulpisut et al., 2012). While ZC is sexually active (Huang et al., 2019), the naturally growing ZZ plants are sexually infertile in China, making it difficult to generate new varieties through sexual crossing. Nonetheless, ZZ can produce mature flowers and gametes with normal structures (Fan et al., 2004). Some ZZ populations in India may produce a small number of seeds (Thomas et al., 2016), implying that ZZ can reproduce sexually. However, it is not clear so far at which step(s) the sexual reproduction in ZZ is inhibited, nor are the regulatory mechanisms that enable this inhibition. To address these issues, we perform a comparison between ZZ and ZC using cytological and transcriptomic techniques, aiming at exploring the major differences and regulations that underlie the sexual abortion of ZZ. The results reveal that most, if not all, invading PTs remain intact and do not rupture in the ovules of ZZ, which is accompanied by large-scale transcriptional suppression. Genes for *ANX1* and *FER*, together with those for the interaction partners or components of the signaling pathway, are regulated in distinct ways in ZZ and ZC, which might be attributed to the non-rupture phenotype and the failure of double fertilization in ZZ.

## Materials and methods

### Plant collection, preliminary treatment, and growth conditions

Samples of *Z. zerumbet* (ZZ) and *Z. corallinum* (ZC) were collected from wild populations on Luofu Mountain (Huizhou, China) and in the town of Lanyang (Danzhou, China), respectively. The stamens of unopened flowers were removed and packaged the day before sampling to prevent natural pollination. At 8:00–9:00 AM of the sampling day, artificial pollination was performed on the opened flowers using pollens collected from other plants. Ovaries were collected from the pollinated flowers immediately (marked as 0 HAP) or at the indicated hours/days after pollination (HAP/DAP).

### Viability assay for pollen grain and stigma

To examine the viability of pollen grains or stigmas, the samples were collected since 9:00 AM and used immediately. The viability of fresh pollen grains was examined using the MTT method (Rodriguez-Riano and Dafni, 2000). Pollen grains were collected from at least five unpollinated flowers, and the number of stained grains in three distinct visual areas for each sample was tallied. The control experiments using heated, inactivated pollen grains were performed in parallel. The viability of unpollinated stigmas was examined using the same method.

### Pollen germination assay

To test the germination competence of pollen on the stigmas, hand-pollination was performed using a pollen grain mixture collected from the same individual plant. Pollinated stigmas were collected at the indicated time points and immediately fixed in Carnoy’s fluid for 12–24 h at room temperature, followed by washing three times in anhydrous ethanol and rehydrating by sequential treatment with 95%, 85%, and 70% ethanol. All samples were stored in 70% ethanol at 4°C until used. The stored pollinated stigmas were rehydrated in a graded ethanol series (50%, 30%, and 15%) and examined under a Leica DM6B microscope. Pollen grains with a pollen tube length equal to or longer than their diameter were considered to have germinated. The germination rate was defined as the percentage of germinated pollen grains versus the total number of pollen grains in a certain stigma. At least five independent stigmas were used to calculate the average germination rate.

### Observation of the PT growth in style and the PT invasion into the embryonic sac

To observe PT growth, styles were collected at different time points after pollination and fixed immediately in Carnoy’s fluid for 12–24 h at room temperature, followed by washing three times in anhydrous ethanol and rehydrating by sequential treatment with 95%, 85%, and 70% ethanol. All samples were stored in 70% ethanol at 4°C until used. These stored styles were rehydrated in a graded ethanol series (50%, 30%, and 15%) and then treated with 5 mol/L sodium hydroxide (NaOH) for 2 h, followed by soaking in distilled water for 1 h. All these specimens were mounted on glass slides and stained with 0.05% aniline blue for 1 h. The stained specimens were covered with a coverslip and squeezed gently. The behavior of PTs in the styles was then observed using the UV channel of a Leica DM6B microscope. The PT growing speed was defined as the average speed during a given period and was estimated by the increased distance (mm) of a cluster of PTs traveled divided by the time it took (hours).

To observe the PT invasion into the embryonic sac, ovaries were collected at different time points after pollination and fixed immediately. The fixed materials were then dehydrated in a graded ethanol series of 85%, 95%, and 100%, followed by permeating and embedding in pure paraffin. Serial sectioning was then conducted using the Reichert Histostat 820 to obtain 5–10 μm-thick sections, which were mounted on glass slices and stained using 0.05% aniline blue for 1 h or 1% hematoxylin for 5 min. Stained samples were covered with a coverslip, and the PTs were observed and photographed under a Leica DM6B microscope in the UV channel (for an aniline blue-stained specimen) or in the light field (for a hematoxylin-stained specimen).

### Observation of the male and female gametophyte development

To investigate the development of male and female gametophytes, anthers and ovaries were collected and fixed in Carnoy’s fluid for 12–24 h at room temperature, followed by washing three times in anhydrous ethanol and rehydrating by sequential treatment with 95%, 85%, and 70% ethanol. All samples were stored in 70% ethanol at 4°C until used. Before the sectioning procedures, the fixed materials were first dehydrated in graded ethanol series of 85%, 95%, and 100%, and then permeated and embedded in pure paraffin. Serial sectioning was conducted using the Reichert Histostat 820 to obtain 5–10 μm-thick sections, which were mounted on glass slices and stained using 1% hematoxylin. The development of gametophytes was observed and photographed with a Leica DM6B microscope.

### RNA-Seq and data analysis

For RNA-Seq analysis, ovaries without styles were collected at the indicated time points after hand-pollination and frozen in liquid nitrogen immediately. All samples were collected in the field after pollination; the frozen materials were then transported to the laboratory and stored at −80°C until needed. Total RNA was extracted from the samples using a WARYONG Quick RNA Isolation Kit (No. 0416-50, HuaYueYang, Beijing, China) according to the user’s manual. All total RNA was sent to Novogene Ltd. (Beijing, China) and quality checked. Samples with RIN ≥8 were selected for RNA-Seq analysis. Library construction, large-scale sequencing using the HiSeq 2500 platform (Illumina), data processing (cleaning, vector trimming, etc.), and preliminary bioinformatics analysis, including unigene assembly, annotation, and expression quantification, were performed by Novogene Ltd. (Beijing, China). RNA-seq data have been deposited in GenBank under accession number PRJNA716061 at https://www.ncbi.nlm.nih.gov/sra/PRJNA716061.

FPKM (fragments per kilobase of transcript per million fragments mapped) values were used to quantify the expression of each unigene in each sample. Unigenes with FPKM differences <2-fold change (FC) between replicated samples and the average FPKM >0.3 at least at one time point were defined as Stable Between replicated Samples (SBS) and were analyzed in our study. Differentially expressed genes (DEGs) were identified under the criteria of fold change relative to 0 HAP ≥2 and an adjusted *P*-value <0.0001. GO enrichment analysis of up and downregulated DEGs was performed using the ClueGO App of Cytoscape_3.7.2 (Bindea et al., 2009). GO annotations of genes in the *A. thaliana* genome were used as a reference. Identification of transcription profiles was performed using the STEM tool (http://www.sb.cs.cmu.edu/stem/).

### Reverse-transcription quantitative PCR

For reverse transcription quantitative PCR (RT-qPCR), 1 μg of total RNA extracted from ovaries was reverse transcribed in a 20-μl reaction volume, and 0.25 μl of the product was used as a template for qPCR in a final volume of 20 μl. Gene-specific primers are listed in **Table S4**. *GAPDH* genes from ZZ and ZC were used as endogenous controls, and expression at 0 HAP was used for calibration. qPCR was performed in an ABI PRISM^®^ 7500 real-time PCR system (ABI, USA), and the relative expression level of each unigene was calculated using the 2^−ΔΔCt^ method (Kenneth and Thomas, 2002).

## Results

### Gametogenesis are similar *in* ZZ and ZC

Preliminary investigations in the field were performed first. The results confirmed that ZZ has well-developed flowers like its congener species ZC, which is completely fertile (**Figure S1**). Nevertheless, the seed-setting frequency was extremely low both in wild ZZ populations on Luo-fu Mountain (Guangdong, China) (<0.5%) and in artificial populations generated from intraspecies crossing (**Table S1**). This agrees with the clonal propagation of ZZ observed in other regions (Thomas et al., 2016) and demonstrated that ZZ is infertile in the tested populations. Mature seeds were generated in interspecific crosses between ZZ and ZC at a frequency of approximately 16% when ZZ served as a pollen donor. However, the inverse cross did not generate seeds (**Table S1**), suggesting that both male- and female-originated regulations account for the sterility of ZZ, although the latter is more plausible.

**Figure 1:**
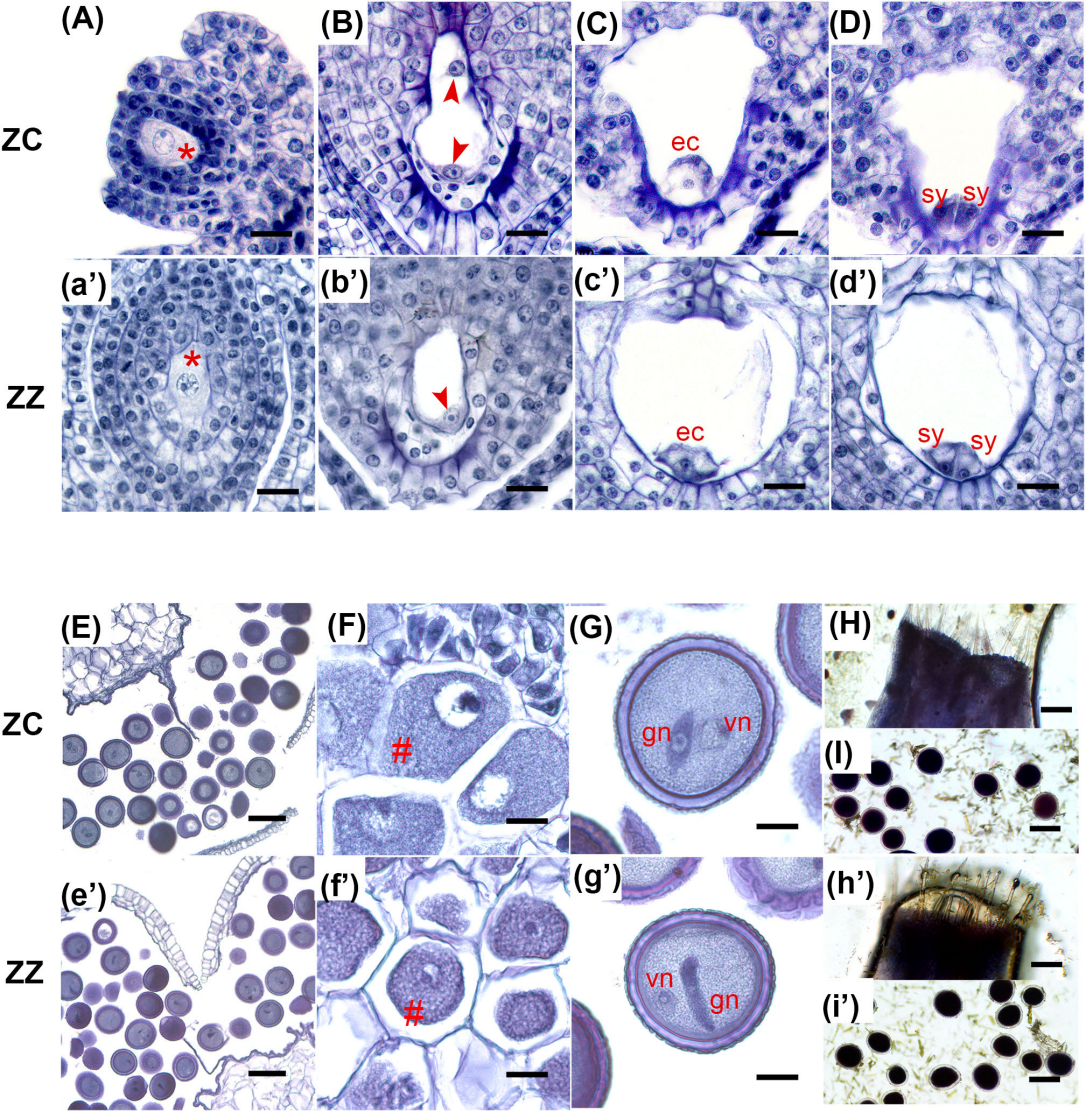
Gametogenesis is similar in ZC and ZZ. **(A–I)** and (a'–i') show characteristic structures during gametophyte biogenesis in ZC and ZZ, respectively, as indicated at left. **(A)**, (a'): The megaspore mother cells (red “*”) in ZC and ZZ are larger than the surrounding cells. **(B)**, (b'): Typical two-nucleate embryo sacs in ZC and ZZ. Red arrowheads indicate the nuclei generated by mitosis of the functional megaspore. **(C, D)**, (c', d'): Two serial paraffin slices of a single mature embryo sac in ZC and ZZ, respectively, showing a well-developed egg cell (ec) (**C**, c') and two synergids (sy) (**D**, d') at the micropyle end. **(E)**, (e'): Transection of a dehisced anther with mature pollen grains in ZC and ZZ, respectively. **(F)**, (f'): Microspore mother cells are indicated by a red “#”. **(G)** (g'): Mature pollen grains with one generative nucleus (gn) and one vegetative nucleus (vn). **(H)**, (h'): Stigma receptivity, as revealed using the MTT method. **(I)**, (i'): Pollen activity, as confirmed by the deep purple color in the MTT assay. ec, egg cell; sy, synergid; gn, generative nucleus; vn, vegetative nucleus. Bars: 20 μm (**A–D**, **F**, **G**, a'–d', f', g'), 100 μm (**E**, **I**, e', i'), 200 μm (**H**, h').

**Figure 2:**
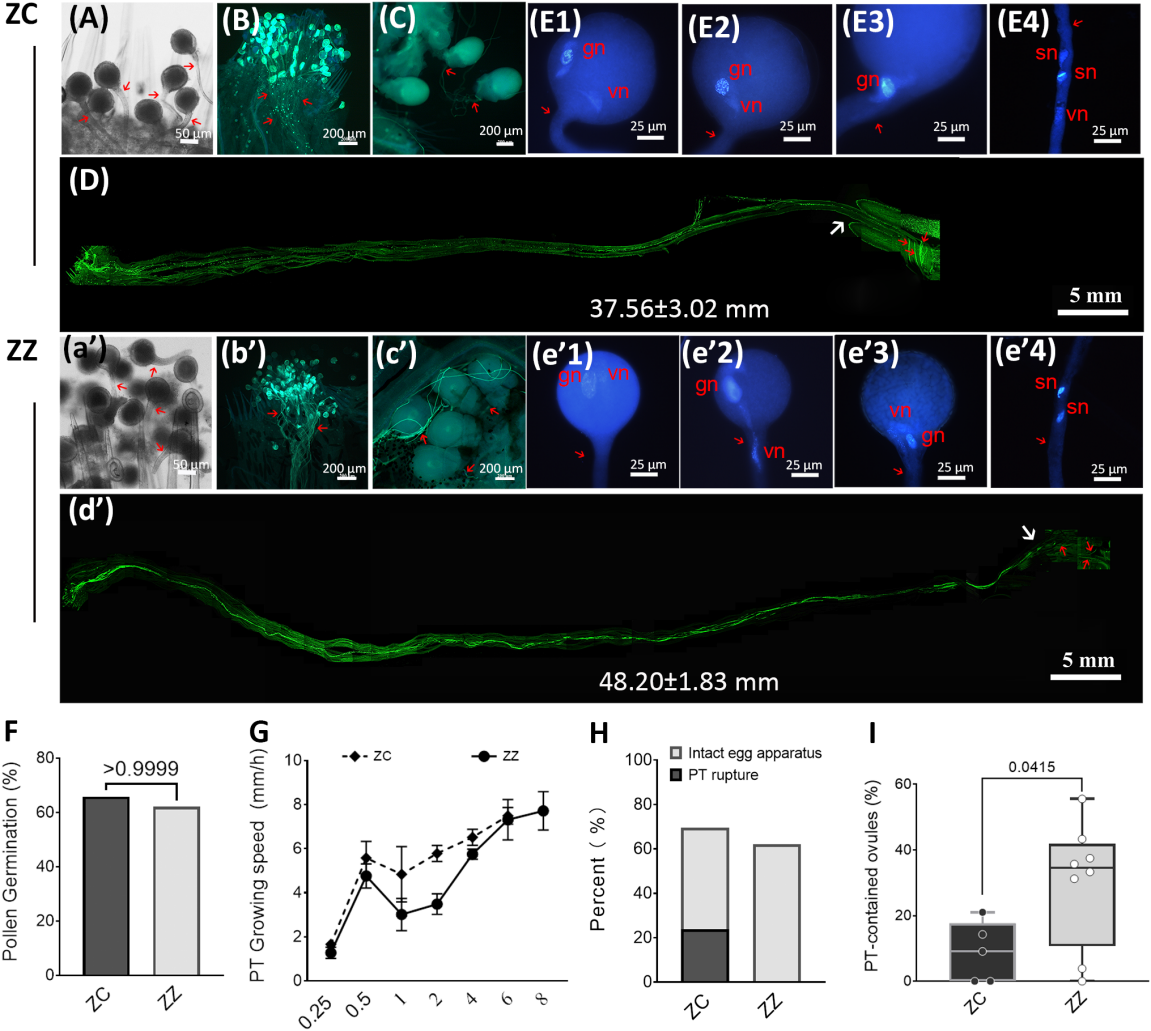
Pollen germination and pollen tube growth are active in ZC and ZZ. **(A–E)** and (a’-e’) show pollen germination and pollen tube growth in ZC and ZZ, respectively. **(A)**, (a’) 15 MAP: Pollen germination on stigmas. Pollen tubes (red arrow) that were longer than the diameter of the pollen grain were observed at 15 minutes after pollination (MAP) in ZC and ZZ, respectively. **(B)**, (b’) 2 HAP: Pollen tubes penetrate across the stigma-style interface and grow along the style at 2 HAP. Red arrows indicate pollen tubes that extended into the style. **(C)**, (c’): Pollen tubes surround the ovules at 12 HAP in ZC **(C)** and at 14 HAP in ZZ (c’). **(D)**, (d’): Pathway of pollen tubes within the style. Images from a single style are shown. A pollen tube cluster can be observed at the entrance of ovaries at 6 HAP **(D)** in ZC and at 8 HAP in ZZ (d’). The white arrow indicates the entrance of an ovary. Red arrows indicate pollen tubes in the ovary. The average length of the styles are shown as mean±SD of 10 samples of each species. Student’s t-test p-value=4.0610-8. **(E1–E4)**, (e’1-e’4): Division of the generative nucleus (gn) in pollen. Pollen grains were germinated *in vitro*: generative nucleus (gn) with condensed chromatin, vegetative nucleus (vn), and two putative sperm nuclei (sn) are indicated. **(F)**: Germination rate of pollen on the stigma in ZC and ZZ. No significant differences existed between ZC and ZZ, as determined by Student’s t-test. **(G)**: Changes in the speed of pollen tube growth in the styles. Average speed (mm/h) = the increased distance of pollen tubes travel (mm)/hour(s). **(H)**: Pollen tube rupture in ZZ or ZC during 12 HAP to 26 HAP. The percentage of ovules with intact egg apparatus or leftovers of rupture pollen tube and destroyed egg apparatus was estimated in a sample of 118 ZC ovules or 129 ZZ ovules. **(I)**: Distribution of the number of embryo sacs containing intact pollen tubes at 24 HAP. A total of 8 ZZ ovaries (158 ovules) and 5 ZC ovaries (77 ovules) were included in the estimation. Student’s t test p-value is shown.

We then asked which steps would be predominantly differential between these two gingers. Using a microscope-based approach, we first evaluated the production and viability of ZZ gametocytes and compared them with ZC. Both species developed typical Polygonum-type female gametocytes (**Figures 1A–D**, a’–d’, **Figures S2**, **3**). Their pollen grains were also well developed (**Figures 1E–G**, e’–g’, **Figures S4**, **5**). Using the MTT method, viability tests indicated that ZZ was able to sustain the viability of both stigmas and pollens till the flowers wilted, which are similar to those of ZC (**Figures 1H, I**, h’, i’, **Figure S6**). Besides, 62.14% of ZZ pollen grains (189/303 pollens from five flowers) were able to germinate on the stigmas, which is also close to the value in ZC (65.84%, 149/228, five flowers) (**Figure 2F**). To observe the production of the generative nuclei in ZZ and ZC, pollens were collected and germinated *in vitro*. The undivided generative nucleus was able to be observed initially in the cell body of a single pollen both in ZZ and ZC, while two sperm cells were visible within the elongating tube subsequently (**Figures 2E1–4, e’1–e’4**).

Shortly after pollination, both ZZ and ZC pollen tubes (PTs) penetrated through the stigma–style interface (**Figures 2A, B**, a’, b’), and extended ahead in the transmitting tract at a high speed during the first 30 min (**Figure 2G**). The changes in the average growing speeds (data from five styles of each species) were similar between ZZ and ZC, and a deceleration occurred after 30 min post-pollination in both species, nonetheless. However, a restoration followed, and the average speeds reached similar values during the final time interval before arriving at the entrance of the ovaries (**Figure 2G**). Tracing of the PT growth revealed that ZZ needed two more hours than ZC to arrive at the entrance of the ovaries (8 HAP for ZZ *vs.* 6 HAP for ZC), which could be attributed to the different lengths of the styles in ZZ and ZC (**Figure 2D, d**’). Nevertheless, a comparable percentage of ZZ PTs (44.16%, averaged from seven ovaries) were observed arriving at the ovaries at 8 HAP as ZC PTs at 6 HAP (38.05%, averaged from four ovaries). Taken together, these observations clearly indicate that the pollens of ZZ are self-compatible, and the emerging pollen tubes can arrive at the ovaries with a similar percentage to that of ZC, suggesting that the sterility of ZZ is not caused by a possible barrier before the PTs grow into the ovaries.

### Higher percent of ZZ ovules contain intact pollen tubes

**Figure 3:**
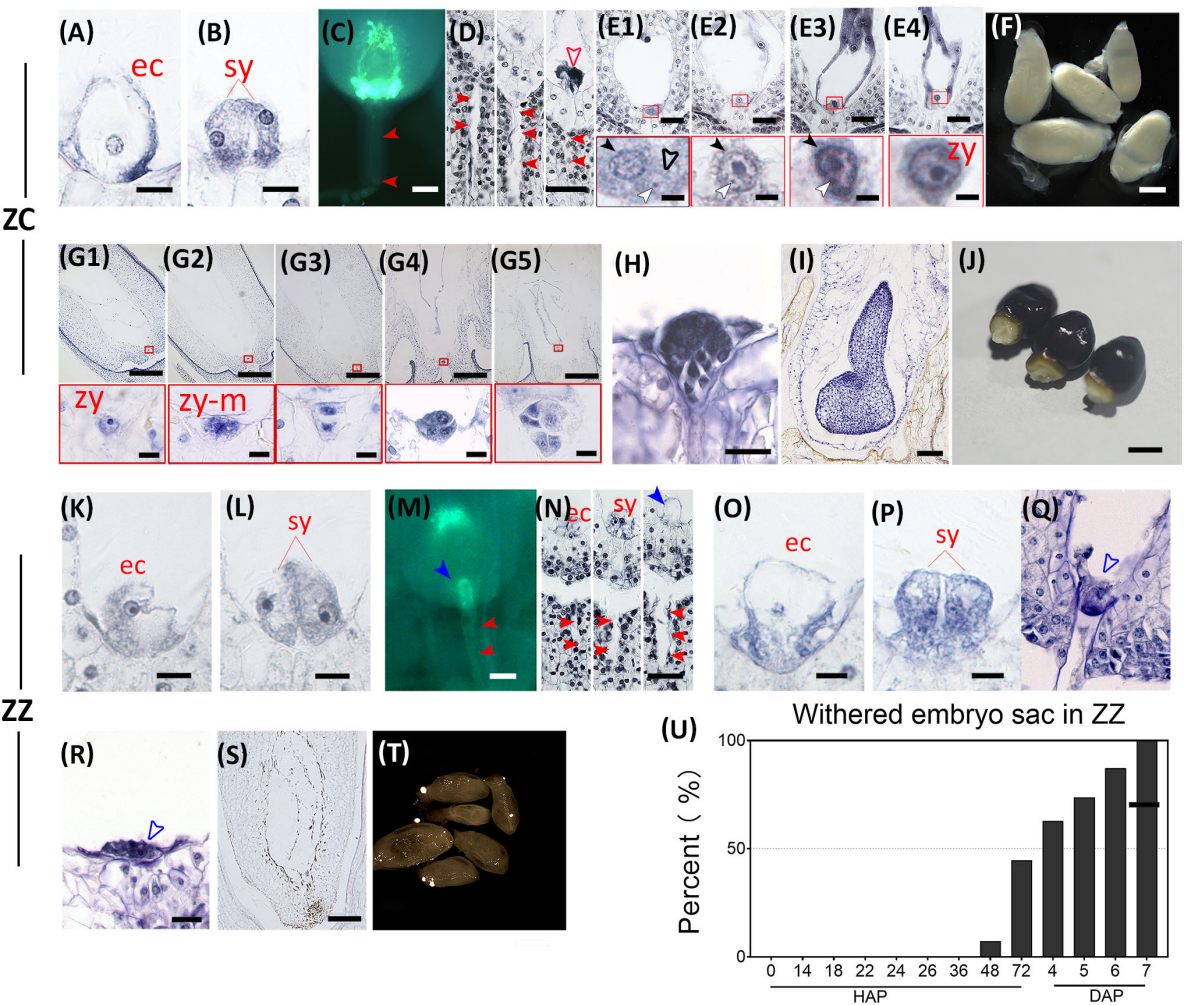
Double fertilization and embryogenesis are functional in ZC but fail in ZZ. Key events at different time points before and after double fertilization are shown for ZC **(A–J)** and ZZ **(K–U)**, respectively. **(A, B)** show the ZC egg apparatus at 0 HAP with a well-developed egg cell (ec) and two synergids (sy). Bar = 10 μm. **(C, D)** show the invading pathway (closed red arrowhead) of pollen tube and the reception of pollen tubes by the embryo sacs at 12 HAP in ZC. The open red arrow indicates the residues from pollen tube burst or degradation of the synergid. Bar = 50 μm in **(C)** and 40 μm in **(D)**. **(E1–E4)** show the fusion progress of the egg cell and sperm nucleus in embryo sacs from 24 HAP to 72 HAP. The red frames show close-up views of the sperm nucleus approaching the egg cell **(E1)**, fusion with the karyotheca of the egg cell **(E2, E3)**, and final fusion of the nucleus to form the zygote **(E4)**. The black closed arrowhead indicates the nucleus of an egg cell; the black open arrowhead indicates the karyotheca of the sperm nucleus; the white closed arrowhead indicates the sperm nucleoli. Bars = 20 μm (above) and = 3 μm (below). **(F)** shows ZC ovules at 7 days after pollination (DAP). **(G1–G5)** show representative embryo sacs observed at 7-17 DAP, respectively. Red frames are close-up views of zygote growth **(G1)**, the first round of mitosis **(G2)**, the two-cell proembryo **(G3)**, and the formation of the multicellular proembryo **(G4, G5)**. Bars = 200 μm (above) and = 10 μm (below). **(H)** shows the representative globular embryo at 1825 DAP. Bar = 25 μm. **(I)** shows shield embryo at 4060 DAP. Bar = 100 μm. **(J)** shows mature seeds at 90 DAP. Bar = 5 mm. **(K, L)** show an ZZ embryo sac containing a well-developed egg cell (ec) and two synergids (sy). Bar = 10 μm. **(M, N)** show the invasion of the pollen tube into the embryo sac at 14 HAP in ZZ. The closed blue arrowhead points to an intact pollen tube in this ovule. Bar = 50 μm in (m) and 40 μm in **(N)**. **(O, P)** show the embryo sac with dissolved karyotheca and nucleoli in the egg cell and synergid at 24 HAP. Bar = 10 μm. **(Q)** shows degenerated egg apparatus (blue open arrowhead at 48 HAP. Bar = 20 μm. **(R, S)** show degraded egg apparatus (blue open arrowhead) and An abortive embryo sac at 6 DAP. Bar = 20 μm in **(R)** and 75 μm in **(S)**. **(T)** shows withered ZZ ovules at 7 DAP. Bar = 500 μm. **(U)** The number of withered ovules increased after artificial pollination in ZZ.

We further traced the PT growth in the ovaries. A few PTs were observed at the base position of the funiculus early at 12 HAP in ZC or at 14 HAP in ZZ (**Figures 2C, c’**, **S7C**). As time passed, a large number were found clustered around the ovules, both in ZZ and ZC. While the majority of PTs wandered outside of the ovules, some were clearly observed inside the ovules (**Figures 3C, D, M, N**, **S7**). In fact, degradation residues of gametophytes could be observed within embryo sacs early at 12 HAP in ZC (**Figures 3A–D**), suggesting that the invaded PTs were ruptured. In comparison, such distinctive features were not found in any ZZ embryo sacs, even after a long time (**Figures 3M, N**). Instead, intact PTs were found in ZZ ovules, and the number of such ovules accounted for an average of 30.07% of total ovules in each ovary in ZZ (eight ovaries, 0.00%–55.56%) at 24 HAP, which was significantly higher than that in ZC (9.14%, five ovaries, 0.00%–21.05%) (**Figure 2I**). We also counted the number of ovules with ruptured PT or with intact egg apparatus over a longer period (from 12 HAP to 26 HAP). The results showed that ZC ovules with ruptured PT accounted for at least 28% of the total ovules, and those containing intact egg apparatus accounted for 54% (**Figure 2H**). Nevertheless, none of the ZZ ovules contained the ruptured PT, and 62.02% of the ovules contained intact egg apparatus (**Figure 2H**). Taken together, these observations indicate that ZZ PTs can invade embryonic sacs, but many of them did not rupture.

Subsequently, ZZ and ZC followed divergent paths. In ZZ, although the appearance of the egg apparatus was maintained even after 24 HAP, the boundary of the nucleus was obscure (**Figures 3O, P**), implying that the nucleus had broken and spilled its contents. After 48 HAP, the egg apparatus had lost its shape, which marked the degeneration of the egg and synergids (**Figure 3Q**). After 6 DAP, the residue of the degenerated egg apparatus was clearly visible under the microscope (**Figure 3R**). In line with this finding, few withered ovules were observed before 48 HAP, but the percentage of the withered ovules increased rapidly from 7.72% at 48 HAP to 100% after 7 days (**Figures 3T, U**). These observations agree with the fact that PTs of ZZ were unruptured in the ovules (**Figures 3M, N, I**). By contrast, sperm nuclei were observed near the egg nuclei in ZC just after 24 HAP, and fusion with the egg was also observed subsequently (**Figures 3E1–4**), which was followed by further embryonic development (**Figures 3G–I**). Taken together, these comparisons between ZZ and ZC indicate that the lack of sexual reproduction in ZZ appears to be due, at least partially, to the failed PT rupture, which prevents successful fertilization.

### Global differences of transcriptional responses to pollination

To identify the primary regulators of the differential PT behaviors between ZZ and ZC, RNA-Seq analysis was performed on the ZZ and ZC ovaries, which were collected at a series of time points. To ensure the analysis focused on transcriptional regulations in relation to the hypothetical ZZ reproduction barrier, only style-removed ovaries were used, which eliminated distractions from the styles. Besides, the time points of 8 HAP or 6 HAP were set as the starting points for ZZ or ZC, respectively, which allow to focus on the events occurring after PT goes into the ovaries. Ovaries at 0 HAP were collected immediately after pollination and were used as control samples for comparison with all other time points. In total, 16 samples of mixed ovaries from ZZ and 17 from ZC were included, comprising two sets of time series for analysis of the transcriptional changes at several vital time points (**Table S2**; **Figure S1B**).

After removing the unreliable reads, *de novo* assembly generated 223,745 and 219,484 unigene sequences for ZZ and ZC, respectively (**Table S3**), and those with a length >1,000 bp accounted for nearly half (**Figure S8A**). GO annotations were assigned to each unigene, and the frequency distribution of the representative GO terms was similar between ZZ and ZC (**Table S3**; **Figure S8C**). The expression level of each unigene was normalized by the FPKM method, and only those with FPKM ≥0.3 at least at one time point were defined as expressed genes. To exclude the noise caused by fluctuations in the natural environment, we performed further screening to choose the so-called SBS unigenes whose expression variations were minor between repeated samples (see the *Materials and methods* section). Regardless of the dataset containing all expressed genes or SBS genes, all distributions of gene counts, FPKM values, and functional classifications revealed no discernible difference between ZZ and ZC (**Table S3**; **Figures S8A–C**). To verify the results of RNA-Seq, RT-qPCR analysis was performed on eight ZZ unigenes and six ZC unigenes, including those proposed to be tightly associated with reproduction, such as *FER* and *ANX1*. The results of RT-qPCR were generally in agreement with those of RNA-Seq (**Figure S9A**). Taken together, these preliminary analyses allowed the comparisons between these ginger species in our study.

### Identifying the differentially expressed genes

**Figure 4:**
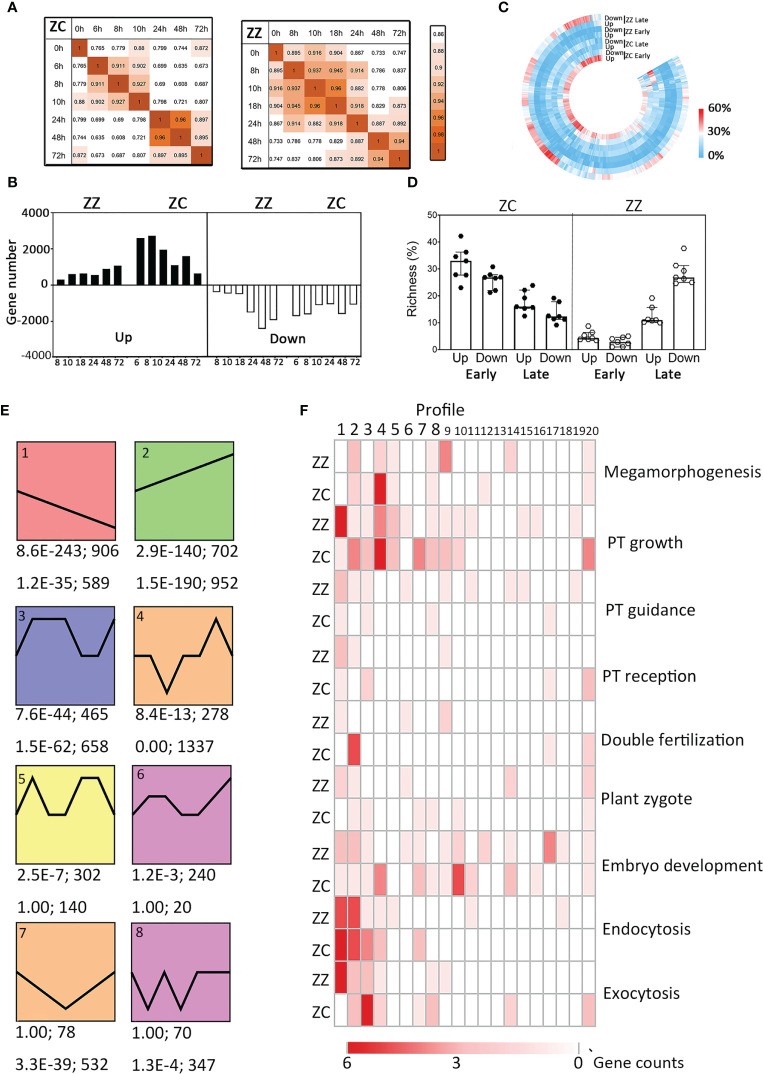
Overview of the global transcriptomic profiling sand differences between ZZ and ZC. **(A)** Stage specific transcription patterns in ZZ (right) and ZC (left). Pearson correlation coefficient (r) were used to identify the time points with similar global transcription. The color depth (orange) indicates the strength of the pairwise similarity. **(B)** The change of the number of DEGs along time in ZZ and ZC. **(C)** Richness of differentially expressed genes (DEGs) involved in each GO biological process (GO-BP) at different stages. Richness is defined as the percentage of DEGs among all genes annotated with a given GO-BP term. Joint GO enrichment analysis was performed as described in *Materials and methods*. Visualization was conducted using TBtools. Each column represents a given GO-BP term, and the color represent the DEG richness as indicated. **(D)** Richness of DEGs involving in seven reproduction-related GO-BP terms. Each symbol represents the DEG richness of a given GO term. **(E)** The representative time-course transcription profiles (Profiles #1–#8) in ZZ and ZC. The p-value and the gene number of a given profile in ZZ (upper) and ZC (down) are shown beneath each panel. **(F)** Richness of DEGs of several reproduction related GO terms in all transcription profiles. The shades of red color indicate the numbers of DEGs.

Pearson product-moment correlation analysis and principal component analysis (PCA) for SBS unigenes were also conducted. The results clearly indicated that the transcriptional responses to pollination of ZZ and ZC can be divided into two phases, namely the early stage and the late stage, which were separated by the time-point of 24 HAP (**Figures 4A**, **S9B**), suggesting specific regulations on these distinct phases.

Differentially expressed genes (DEGs) were further identified from the SBS unigene set based on the fold change in expression relative to 0 HAP and the corrected *p*-value, generating a total of 5,580 and 8,627 DEGs for ZZ and ZC, respectively. The number of DEGs increased with time in ZZ while decreasing in ZC (**Figure 4B**), suggesting a delayed induction of global transcription in ZZ. GO enrichment analysis also revealed distinct profiles of functional gene classes. A large proportion of GO-biological processes that were early upregulated in ZC were not disturbed at the same stage in ZZ but showed downregulation patterns at the late stage (**Figure 4C**). In particular, the DEG richness of seven reproduction-related GO-BPs decreased with time in ZC but increased in ZZ (**Figure 4D**), in agreement with the suggestion of the untimely activation of reproduction processes in sterile ZZ species.

**Figure 5:**
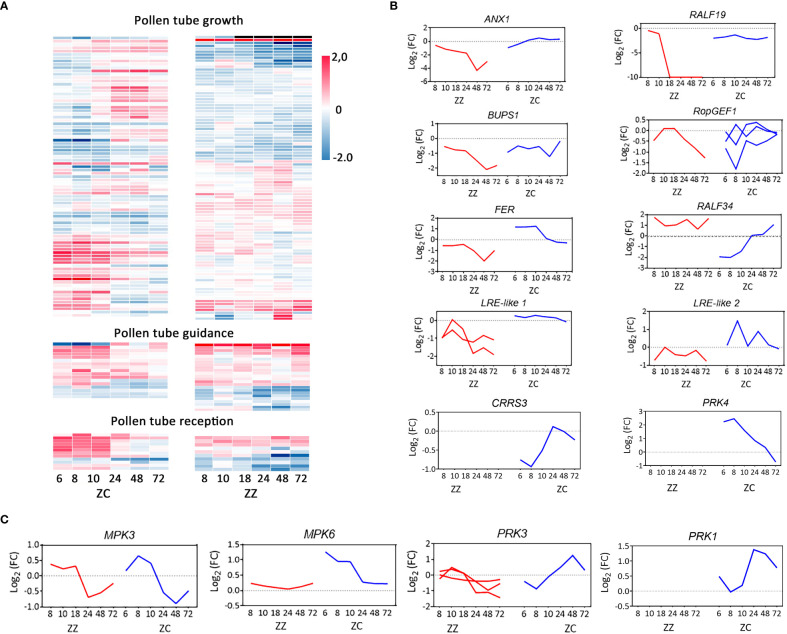
Differential regulations on pollen tube growth, guidance, and reception in ZZ and ZC. **(A)** Heatmaps of the time-course expression. Unigenes annotated involving in the GO-biological processes of pollen tube growth, guidance and reception are included in hierarchical clustering analysis. Log2 (fold change relative to 0 HAP) of all genes were used as input data. **(B, C)** Time-course transcriptional changes of master regulators of pollen tube growth, guidance, and reception.

To further get insight into the differences in transcriptional regulation of sexual reproduction, we identified the top 20 representative transcript profiles enriched by DEGs using STEM (http://www.sb.cs.cmu.edu/stem/) (**Figures 4B**, **S10**). Interestingly, all 20 profiles were shared by ZZ and ZC, but they were ranked differentially based on the statistical *p*-value. DEGs involved in biological processes, including those in relation to PT growth, guidance, and reception, were differentially distributed in these profiles (**Figures 4E, F**). In the ZZ gene set of Profile #1 that displayed a decreasing tendency with time, twelve genes were annotated as involving PT growth, accounting for 41.38% of the total PT-associated DEGs (**Figure 4F**). A similar bias in the decreasing pattern of Profile #1 was also found in ZZ DEGs in relation to PT guidance and reception, as well as those in relation to plant zygote and embryonic development. ZC was very different and displayed preference for the upward regulations (e.g., Profile #2) or for the transient downregulations that were followed by rapid recovery (e.g., Profile #4). Notably, the expression heatmaps indicated stage-specific regulations in ZC, while they were likely lost in ZZ (**Figure 5A**). Altogether, these findings suggest differential transcriptional regulations on reproductive processes in ZZ versus ZC.

### Differential regulations on PT growth

In total, 98 genes from ZZ and 85 genes from ZC were recognized to be involved in PT growth (**Table S5**), among which 29 (29.59%) and 36 (42.35%), respectively, were differentially regulated relative to 0 HAP (**Figure S6**). The PT growth-related DEGs of ZZ biased Profile #1, which displayed a time-dependent decreasing tendency (**Figures 4E, F**), and accounted for 41.38% (12/29) of the total PT growth-related DEGs of this species (**Figure 4F**). In comparison, only 2.78% (1/36) of ZC PT growth-associated DEGs were involved in this profile, but 25% (9/36) were distributed in Profile #4, which was the most significantly over-representative profile in ZC (*p*-value = 0) and presented a dramatic downregulation at 8 HAP but recovered rapidly and peaked at 48 HAP (**Figure 4E**). Profiles #2, #7, and #20 each contained 11.11% (4/36) of ZC DEGs, versus 3.45%, 0.00%, and 0.00% in ZZ (**Figure 4F**).

From Profiles #1 and #2, the profiles with completely opposite patterns, a pair of orthologous DEGs (ZC_Cluster-39943.147126/ZZ_Cluster-6389.104551) (**Figure S11A**), which coded for receptor-like kinases in prediction, were recognized. The deduced proteins of these genes shared high identities with *Arabidopsis* ANXUR1 (AtANX1 protein) (>58%) and were named ZC_ANX1 and ZZ_ANX1, respectively. Notably, *ZC_ANX1* and *ZZ_ANX1* were regulated in opposite manners in ZZ and ZC, increasing in ZC but decreasing in ZZ (**Figure 5B**). The overall amino acid identity is up to 98% between ZC_ANX1 and ZZ_ANX1, whereas the amino acid substitution between them is rich in the extracellular domain compared to the predicted cytoplasmic region (12 vs 5 sites) (**Figure S11B**), implying differential capacity of these proteins for signal reception. What is worth noting is that a gene (Cluster-6389.61957) encoding Buddha’s Paper Seal1 (BUPS1), which forms a complex with the ANX1 protein on the plasma membrane of PT and is critical for PT growth and integrity (Zhu et al., 2018), was co-downregulated in ZZ. In ZC, however, the *BUPS1* homologous gene maintained a constant level throughout the entire period the PT traveled inside the ovaries (**Figure 5B**). The other pair of DEGs identified from Profiles #1 and #2 (ZC_Cluster-39943.135905/ZZ_Cluster-6389.75179) code for ROP guanine nucleotide exchange factor 1 (RopGEF1), which can physically interact with BUPS proteins (Zhu et al., 2018) and serve as an activator of ROP (Rho of Plants) GTPases during PT growth (Gu et al., 2006). Similarly, the expression of *ZC_RopGEF1* followed an increasing trend, whereas that of *ZZ_RopGEF1* was downregulated with time (**Figure 5B**). Taken together, these results suggested that transcriptional regulation on the ANX1/BUPS1 complex, as well as the downstream signaling mediated by RopGEFs, would be suppressed in ZZ but tend to be active in ZC.

We then asked whether the upstream components of this ANX1/BUPS1-RopGEF1 signaling pathway were cooperatively regulated. We recognized a sequence from ZZ for the predicted rapid alkalinization factor 19 (RALF19), a peptide ligand of the ANX/BUPS receptor complex (Johnson et al., 2019). Expression of this gene follows the Profile #1 pattern in ZZ, keeping pace with ZZ *ANX1* and ZZ *BUPS1*. It was initially downregulated at 8 HAP and completely disappeared after 18 HAP (**Figure 5B**). The ZC homologs were also downregulated at 6 HAP but maintained a constant low level afterwards (**Figure 5B**; **Table S9**). Sequence alignment indicated that a signal peptide was lost in the predicted ZC_RALF19 (**Figure S12**). Besides, a total of 14 substituted sites were found in the mature sequence (52 amino acids) of ZC_RALF19 compared with ZZ_RALF19 (**Figure S12**).

It was noted that the predicted *LRX8* gene, whose *Arabidopsis* homolog can help stabilize the ANX/BUPS-RALF19 complex (Mecchia et al., 2017; Fabrice et al., 2018), was co-expressed with *RALF19*, *ANX1*, and *BUPS1* in ZZ (**Table S9**). These and the above results strongly suggested that expression of the RALF19-LRX8-ANAX1/BUPS1-RopGEF1 module was suppressed to a low level in ZZ, and the PT integrity in ZZ would be lost after 18 HAP due to the disappearance of transcription of *RALF19* and *LRX8*.

### Differential regulations on PT reorientation

Four PT guidance associated DEGs of ZC were identified from the DEG dataset of all 20 profiles, accounting for 23.53% (4/17) of the associated SBS unigenes. The DEG percentage in ZZ was 45.83% (11/24), approximately double that of ZC, suggesting a higher level of transcriptional change or disruption of PT guidance in ZZ. We then performed pairwise comparisons for orthologous DEGs between species.

We firstly focused on the funicular guidance-related *MPK3* and *MPK6* (Guan et al., 2014). The ortholog relationship of unigenes from ZZ and ZC was clarified based on the phylogenetic relationship (**Figure S13A**). Transcription comparison between *MPK3* orthologs (ZC_Cluster-39943.89036/ZZ_Cluster-6389.93699) indicated that they were regulated in a similar way in ZZ and ZC, showing activation before 24 HAP and a decrease after that (**Figure 5C**). The Cluster-39943.101002 for ZC_*MPK6* was transcribed at a higher level after pollination, particularly at the early stage. That of the ZZ_MPK6, however, was not changed along with time extending (**Figure 5C**). These results suggest that funiculus guidance in relation to the MPK protein would be similar between ZZ and ZC, although it could be more active in ZC.

We then checked the transcriptional changes of PRK proteins, which were reported to be related to micropylar guidance (Takeuchi and Higashiyama, 2016). Intact ORFs coding for PRK3-like proteins were identified in ZZ and ZC. *ZC_PRK3* was slightly downregulated at the early stage but was upregulated afterwards, presenting an overall increasing pattern (**Figure 5C**). Its closest ortholog in ZZ, the Cluster-6389.154265, was not frequently disturbed (**Figures S13B**, **5C**). DeepTMHMM (https://dtu.biolib.com/DeepTMHMM) (Hallgren et al., 2022) predictions revealed a typical signal peptide located at the N-terminal of ZC_PRK3, followed by a segment for the extracellular region and another for the transmembrane region, all of which were lost in the ZZ_PRK3 protein, implying defection in attractant signal reception by PRK3 in ZZ and probably impairing the PT reorientation (**Figure S13C**). A partial ORF for PRK1 was also found in ZC, which was regulated cooperatively with ZC PRK3 (**Figure 5C**). Notably, the transcript of ZC_PRK4 was regulated with an opposite trend to that of ZC_PRK1/3 (**Figure 5B**), suggesting different roles for these proteins. Orthologs of PRK1 and PRK4 were not identified from ZZ in the present study.

We then attempted to identify transcripts that probably coded for attractant peptides that would be received by PRKs. By computing the Pearson correlation coefficient with the PRK proteins, a ZC DEG coding for a peptide similar to *Arabidopsis* cysteine-rich repeat secretory protein 3 (*ZC_CRRS3*) was found to co-express with *ZC_PRK3* with a correlation coefficient of up to 0.91 (*p*-value = 0.012) (**Figure 5B**). However, none of the ZZ SBS genes for cysteine-rich repeat secretory proteins was identified as the ortholog of the *ZC_CRRS3* gene. Correlation coefficients of the *ZZ_PRK3* ortholog to genes for cysteine-rich repeat secretory proteins were also computed, but none of the candidates was found to be highly co-expressed with *ZZ_PRK3*. Taken together, these results suggest that micropylar guidance for PT reorientation in ZC would be activated through gradually increasing the gene expression of PRK3/PRK1 and their involved signaling cascade, whereas these mechanisms could be suppressed or lost in ZZ.

### Differential regulations on the PT reception

Dramatic differences were also found in the DEG group in relation to PT reception. Profile #1 with a decreasing trend contained 60% (3/5) of the PT reception-related DEGs in ZZ versus the 14.29% (1/7) of ZC DEGs (**Figure 4F**). Additionally, the observation of intact PT within the embryo sacs of ZZ (**Figure 3**) also suggested that a portion of PTs would ignore the suppression of the RALF19-LRX8-ANAX1/BUPS1-RopGEF1 module and invade the ovules. However, no PT discharge was observed in ZZ, in our experience, even after a long time. One possible cause for this phenomenon would be the failure of the PT–synergy interaction. We then screened for those genes involved in the regulation of two complexes, the ANX/BUPS and the FER/LRE complexes, which are essential for this cell–cell interaction. Analysis of the ANX/BUPS can be found in the above sections. Several unigenes for FER homologs, the critical component of the FER/LRE complex, were identified in ZZ and ZC (**Table S9**). Among those from ZC, three unigenes (Cluster-39943.91203, Cluster-39943.92403, and Cluster-39943.94003) were upregulated (Profile #3 or Profile #20), while the remaining one (Cluster-39943.94002) was continuously downregulated (Profile #1 pattern) like the two ZZ *FER* homologs (Cluster-6389.101417 and Cluster-6389.105224) (**Figure 5B**). Importantly, the orthologous pair of *FER* genes from these two species (**Figure S11A**) followed opposite regulations, displaying early activation followed by calming down in ZC versus no-alteration at the early stage and suppression at the late stage in ZZ (**Figure 5B**). We also identified the unigenes that encoded the LRE-like proteins (**Table S9**). These genes were downregulated in ZZ and maintained at a low level (**Figure 5B**). However, those for ZC LRE homologs were dramatically upregulated before 24 HAP and recovered to the normal level after that (**Figure 5B**). Importantly, expression of these genes was in accordance with their respective *FER* partners, suggesting cooperative regulation of the complex they involved in each species (**Figure 5B**).

The repressions of the ZZ genes that encode the components of the ANX1/BUPS1 complex and the FER/LRE complex strongly suggest that they were regulated in a way different from that in ZC. We then asked whether the putative ligands for these kinase–receptor complexes were changed cooperatively to eventually execute differential propagation tasks. RALF34 was previously reported to act as the ligand for this purpose (Ge et al., 2019a). The unigenes for RALF34 were identified in ZZ and ZC. Mature peptides of the predicted RALF34 were almost identical in these species (**Figure S12B**). Nevertheless, the expression of *RALF34* homologs was different in ZC versus ZZ. The predicted ZC *RALF34* was initially downregulated at 6 HAP but rapidly increased after this time point by about 3.5-fold at 24 HAP and close to 10-fold at 72 HAP (**Figure 5B**; **Table S9**), which would provide sufficient RALF34 peptides to support the PT–synergy interaction at the right time. However, ZZ *RALF34* maintained a relatively high level of expression throughout the entire time period (**Figure 5B**), which disagreed with the requirement for timely regulation for successful PT reception.

Other RALF candidates expressed in ZZ and ZC ovaries were identified as well. For example, the homologs of *RALF22* and *RALF33* were found in both species. The predicted ZZ *RALF33* unigene was expressed in an increasing trend, while the ZC *RALF33* was expressed in the opposite trend (**Table S9**). Notably, the predicted ZZ *RALF1* was specifically expressed in ZZ, following the trend of Profile 1, and the predicted *RALF23* was specifically expressed in ZC and followed a decreasing trend after early induction at 6 HAP (**Table S9**).

## Discussion

### PT growth, reorientation and rupture are cooperatively activated in ZC

In *Arabidopsis*, ANXUR proteins form a receptor-like complex together with BUPSs, LLGs, or LRXs, promoting PT growth and maintaining PT integrity by binding the RALF4 or RALF19 signal peptide or promoting sperm discharge by transmitting the RALF34 signal at the moment of PT–synergid interaction (Ge et al., 2017; Fabrice et al., 2018; Zhu et al., 2018; Feng et al., 2019; Ge et al., 2019b). Our transcriptomic data revealed a continued reactivation of the *ZC_ANX1* and a comparatively constant profile of the *ZC_BUPS1* during the PTs travel inside the ovaries (**Figure 5B**). These findings, together with the simultaneously increased expression of *ZC_RopGEF1*, suggest an active signaling module of “ANX1/BUPS1-RopGEF1,” which agrees with the observations that ZC PT is growing smoothly and maintaining integrity in this species (**Figures 2**, **3**). Accordingly, the ZC_RALF19 homolog, a putative upstream signal peptide of the “ANX1/BUPS1-RopGEF1” module, was co-expressed and sustained its transcript level during the whole time (**Figure 5B**), which would continuously provide the signal to the developing PT. It is noted that, however, the predicted ZC_RALF19 protein lacks the signal peptide (SP) (**Figure S12**), which hints that it needs help from unknown transport companion(s) to target the outside of the cell or would stay inside the cell to function as an intracellular ligand of other receptors or complexes. The activation of exocytosis at 6–10 HAP (**Figure 4F**) in ZC would provide an opportunity for RALF19 or other signal peptides to be transported out of the cell. Nevertheless, another attractive hypothesis is that this SP-lacking RALF19 would serve as an intracellular signal for those cytoplasmic RLKs of the *Catharanthus roseus* RLK1-like (CrRLK1L) subfamily, such as MARIS (Boisson-Dernier et al., 2015). If this is true, other members of the RALF family would take the place of RALF19 to stimulate the “ANX1/BUPS1-RopGEF1” signaling pathway. In-depth studies in the future will be required to seek the answers.

The RALF34 was reported to secrete from the female tissue in *Arabidopsis and* serve as a paracrine signal to induce PT rupture by outcompeting the RALF4/19 on the ANX/BUPS complex (Ge et al., 2019a). The *ZC_RALF34* gene was reactivated after an early downregulation (**Figure 5B**), which would be beneficial for the timely induction of PT rupture and avoid premature bursts of PTs before they invade embryonic sacs. On the side of the synergid cell, the early activation of the *ZC_FER* gene since 6 HAP (**Figure 5**) implies an in-advanced accumulation of the LRE/FER complex on the membrane, preparing for the receiving of RALF34 to induce the subsequent synergid degradation and sperm cell discharge (Escobar-Restrepo et al., 2007). Taken together, our findings suggest that ZC_RALF34 would serve as the female-originated key regulator of double fertilization in ZC under the prerequisite of high accumulation of the FER/LRE complex on the membrane of the synergid cell.

### Arrest of pollen tube in ovules is associated with sexual infertility of ZZ

Sexual reproduction in plants is influenced by many factors, and the reproductive barriers result from improper regulations on any steps at the stages of pre- and post-fertilization. Although ZZ adopts clonal propagation in many populations, successful sexual reproduction was documented in a few populations in India (Thomas et al., 2016). The findings of our current study give support to the sexual potential of the species, which stems from their well-developed and active gametophytes (**Figures 1**, **2**, **S1**, **S3**, **S5**, **S6**). Nevertheless, the wilting or abortion of ZZ embryo sacs, the inability to develop zygotes, and the rarity of seed-setting in plants in the garden or in the field (**Figure 3**; **Table S1**) all clearly indicate that sexual reproduction in this species can be blocked. To a large extent, the barrier(s) is not caused by the non-functioning of pollens or stigmas since the ZZ pollens can germinate on the stigmas and the PTs can penetrate the style. However, PT growth and guidance would be impaired since transcription of genes related to these events were dysregulated in ZZ, presenting transcriptional suppression on the RALF19-ANX1/BUPS1-RopGEF1 signal cascade and the ZZ_PRK3 (**Figure 5**). Nevertheless, the fact that the emerging PTs were able to eventually arrive at ovules (**Figures 2**, **3**) and that a large number of PTs were found inside the embryo sacs of ZZ at 24 HAP suggested that the path for ZZ PTs traveling from the stigma to the ovule has not been entirely blocked in this species and will permit a portion of PT to invade the embryo sacs eventually. However, the fact that a higher percentage of ZZ ovules contained intact, or unruptured, PTs and no PT burst was observed in our ZZ samples, which is quite different from what occurs in ZC (**Figure 3**), strongly suggested the existence of a barrier before the occurrence of double fertilization.

### The programmed regulations on the PT reception in ZZ

A transcriptomic study based on the time-course changes inside the ovaries revealed dysregulations in the putative genes for the PT-harbored ANX1/BUPS1 complex and the synergid-harbored LRE/FER complex, suggesting that the PT reception by the synergid would be impaired in ZZ. On the side of PT, the rapid downregulation of genes for the ANX1/BUPS1 complex and its putative upstream signal ZZ_RALF19 (**Figure 5**) after the 18 HAP would be in relation to a predicted slowdown of PT growth, a phenomenon that was reported for the *Arabidopsis* PT after invading the embryonic sac (Johnson et al., 2019). Unfortunately, we did not observe such a slowdown in the present study. Instead, another possibility is that this downregulation would be a programmed mechanism for shutting down the RALF34-activated ANX1/BUPS1-signaling under the high level of *ZZ_RALF34* transcription to avoid the RALF34-induced premature PT rupture as far as possible and maintain the PT integrity during PT growth in the transmitting tract. On the other hand, the shutting down mechanism is also a reasonable explanation for the unruptured phenotype of PTs even after they invade the embryonic sac (**Figures 2H, I**, **3**).

On another side of the PT–synergid interaction, the dramatic transcriptional suppression on the LRE/FER complex led to speculation that only a small number of LRE/FER complexes were able to target the synergid membrane, which may definitely impair the synergid’s ability to receive the PT. One cause for this impairment is the sharp decrease in ZZ_FER yield due to the suppression of gene expression after 18 HAP (**Figure 5B**), while another is that the transport of ZZ_FER protein to target the membrane might be attenuated as well by the low production of LRE-like proteins (**Figure 5B**), the possible transport companion of ZZ_FER if they act like their *Arabidopsis* homologs (Li et al., 2015; Li et al., 2016). Thus, it can be expected that the plasm membrane-anchored FER/LRE complex would be insufficient to support RALF34-mediated signaling, which would subsequently impair the PT rupture in the embryo sac. Taken together, the transcriptional suppression of these complexes that are critical for PT–synergid interaction points out a possible barrier that can block the burst of PT and the release of sperm cells in ZZ embryo sacs.

It should be noted that the infertility of ZZ could be female-derived to a somewhat large extent based on the seed-setting data from the cross experiments (**Table S1**). The findings of the suppression of female-originated FER/LRE complexes and RALF34-mediated signaling can provide support for this hypothesis. Besides, the constant expression of the putative upstream signal ZZ_RALF34 gives two hints: one is that at least some PTs would be prematurely ruptured before they invade the embryo sacs, and another is that some other PTs might be able to rupture successfully inside the embryo sac. However, both of these two hypotheses cannot be verified in our cytological experiments. The low production of the FER/LRE complex and the inefficient anchorage of this complex on the synergid plasm membrane may reject the latter hypothesis of PT rupture in the embryonic sac. However, it is unclear whether the former hypothesis is true. Well-designed and in-depth studies are required to verify it in the future. Taken together, the findings in the present study put forward that the invading PTs, if not all, fail to be ruptured in the infertile species ZZ, which might result from transcriptional suppressions on both the PT-harbored ANX/BUPS complex and the synergid-harbored FER/LRE complex and the shut-down of the relevant signaling in the male and female cells.

### Conclusions and challenges

**Figure 6:**
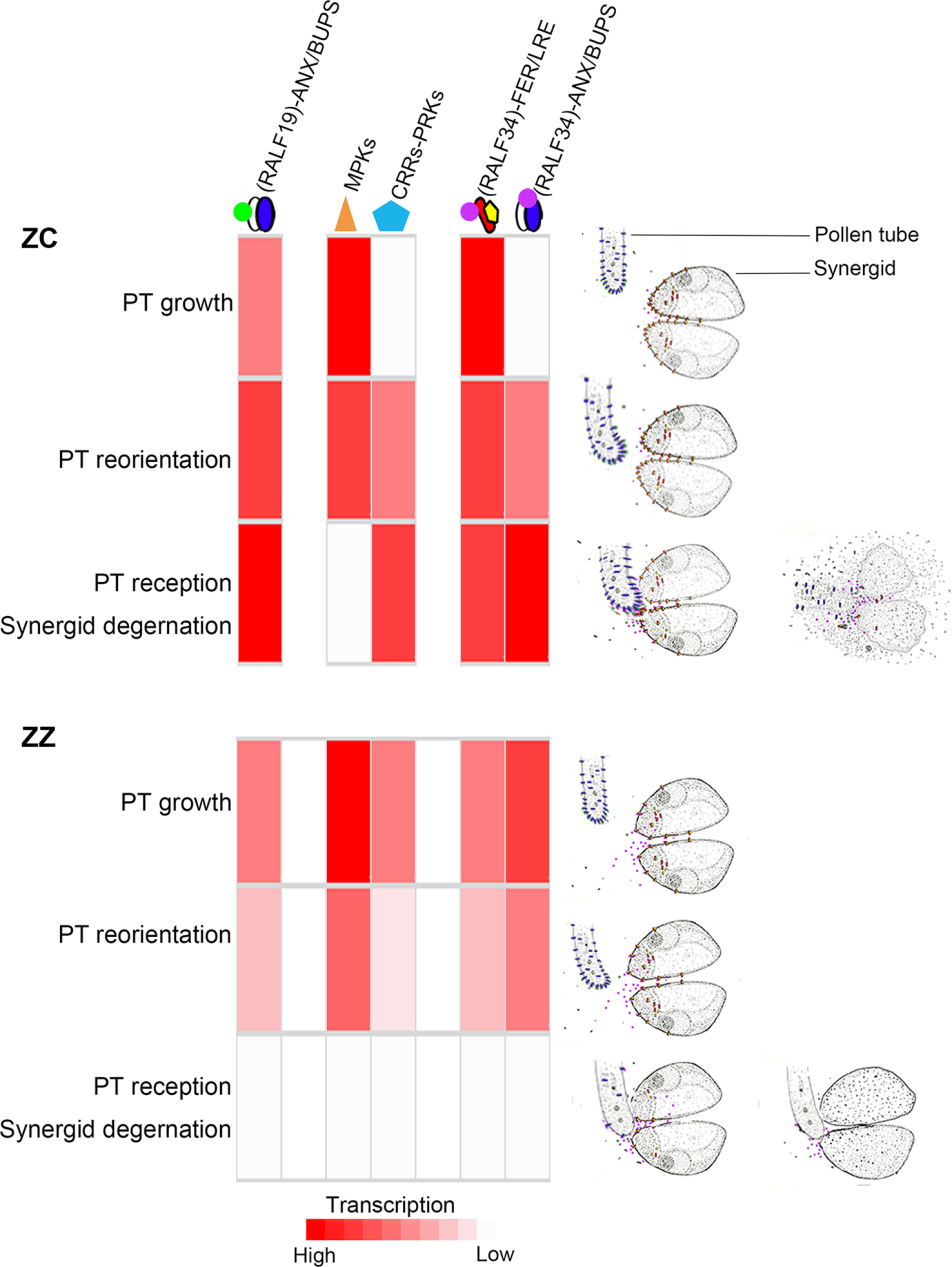
The hypothesized regulation model in ZZ and ZC. The transcription level of genes for the proposed master proteins or complexes are indicated by the shades of red color, which were inferred based on the relative expression at each stage. Although differences in amino acid sequences were observed between ZZ and ZC, they were not included in this model since the implications of these variations are unknown. The appropriate PT and synergids actions are displayed to the right. In brief, ZZ and ZC would have developed distinctive mechanisms to control pollen tube reception. In ZC, the fertile species, the continuously active expression of the ANX/BUPS-RopGEFs signaling module, as well as the timely activated expression of the MPKs and PRKs, ensure the pollen tube growth and reorientation to eventually arrive at the ovules and invade the embryo sacs, where the pollen tube could be received by the active synergid. At this point, the increasing transcription of *RALF34* could provide the signal peptides that would be recognized by the synergid cell’s pre-existing FER/LRE complex or the pollen tube’s abundant ANX/BUPS complex, stimulating pollen tube rupture and sperm cell discharge. Although gene expression of these proteins or complexes would be activated early in ZZ for pollen tube growth and guidance, allowing some pollen tubes, but not all, to arrive at the ovules, these pollen tubes would be prevented from efficient interaction with synergid due to the low production of the ANX-BUPS complex and the FER-LRE complex, as well as the low efficiency of docking to the membrane.

In summary, we propose regulation models for ZZ and ZC, respectively, that they use to programmatically control the behaviors of the PTs and synergids (**Figure 6**). In ZC, genes for the master proteins or complexes of the pre-fertilization events could be timely activated, which would promise the growth, reorientation, and final reception of the pollen tubes. During PT growth inside the ovaries, the signaling cascade RALFs-ANX/BUPS-RopGEFs would be continuously activated, probably due to the increased expression of *ANX*. The early activation of *MPK3* and *PRK3/PRK1* ensures the redirection of the PTs towards the ovules, where they would be received by the embryo sacs for the subsequent sperm cell release. The successful reception of PT by the synergid in ZC would be the consequence of the transcriptional cooperation of the signal peptide RALF34 and the in-ready accumulation of the FER/LRE complex on the plasm membrane of the synergid, which would allow the rapid and efficient transmission of the RALF34 signal and induce the rupture of PT and the discharge of sperm cells in the embryo sacs.

It seems that the sterile ZZ develops a different program to regulate these steps. In this species, the double fertilization would be blocked step by step. At the first step, genes for the components of the ANX/BUPS-RopGEFs module and its ligands, such as RALF19, were rapidly downregulated, which would affect PT growth and lead to premature rupture of PT to some extent. At the second step of PT guidance, those genes for the MPK3 or PRK3/PRK1 proteins were also downregulated after a weak early induction, also suggesting a suppression of PT guidance to the ovules. Blocks from these two steps were leaky and could allow some PTs to eventually reach the ovules and invade the embryo sac. The third step would serve as the final barrier for blocking the reception of PT by synergid, which is probably derived from the suppression of both the ANX/BUPS complex and the FER/LRE complex and may be the key to achieving sterility.

To further discover the mechanism of the sterility of ZZ, it needs to identify the upstream factors that allow the repression of the *ANX1* and *FER* genes. The data in our present study provide a source for exploring the candidates in the future. Besides, the well-developed male and female gametes in ZZ suggest the capacity for sexual reproduction in this species. To create novel cultivars through intra- or inter-species crosses, the removal of the reproduction barriers is required, and this relies on the understanding of the mechanisms underlying fertility. Findings in our study may provide clues for this purpose.

Asexual propagation is a universal strategy that is employed throughout the plant kingdom. Many non-model plant species represent excellent systems for exploring the genetic and evolutionary mechanisms underlying the selection of reproductive strategies. While the findings in our present study provide a possible model to explain the failure of sexual reproduction in ZZ, no clues for the activation of clonal propagation in this species have been hunted. Well-designed experiments focusing on this issue are required.

## Data availability statement

The datasets presented in this study can be found in online repositories. The names of the repository/repositories and accession number(s) can be found below: https://www.ncbi.nlm.nih.gov/sra/PRJNA716061.

## Author contributions

SL contributed to experiment design, analysis of RNA-Seq data and article writing. M-lH performed and analysis the cytological experiments. H-cL and P-pP performed the RNA-Seq experiments and some cytological experiments respectively. H-jH cloned the candidate genes. G-hL and X-pN collected plant materials in field and performed cytological analysis. S-lS contributed to analysis of RNA-Seq data. X-jW participated the experiment design (RNA-Seq) and article review. Y-qW participated the experiment design (field study), population data analysis and article review. HW designed and supervised the whole project and article writing and revision. All authors contributed to the article and approved the submitted version.
