# Supplementary material for: Transcriptional regulations of pollen tube reception are associated with the fertility of the ginger species Zingiber zerumbet and Zingiber corallinum
Source: Front Plant Sci. 2023 May 10;14:1099250. doi: 10.3389/fpls.2023.1099250 (PMC10208065; doi:10.3389/fpls.2023.1099250)

Figure S1. Flower organs of ZZ. (a). Flower organs of ZZ. **(b).** Carpel diagram and the sampling scheme in this study. Time points mark the major behaviors of pollen tubes and ovules. For example, at 8h pollen tube arrives at the entrance of ovary and less are found in ovules, whereas at 10h -12h pollen tube can be observed inner the ovules in ZC. Ovaries were collected by removing styles at time points indicated in red. All samples are mixture of at least 3 ovaries and 2-3 replicates are set up for each time point. Total RNA are extracted from each independent sample for RNA-seq analysis. The stages are defined by integrative results from cytological study and RNA-seq.

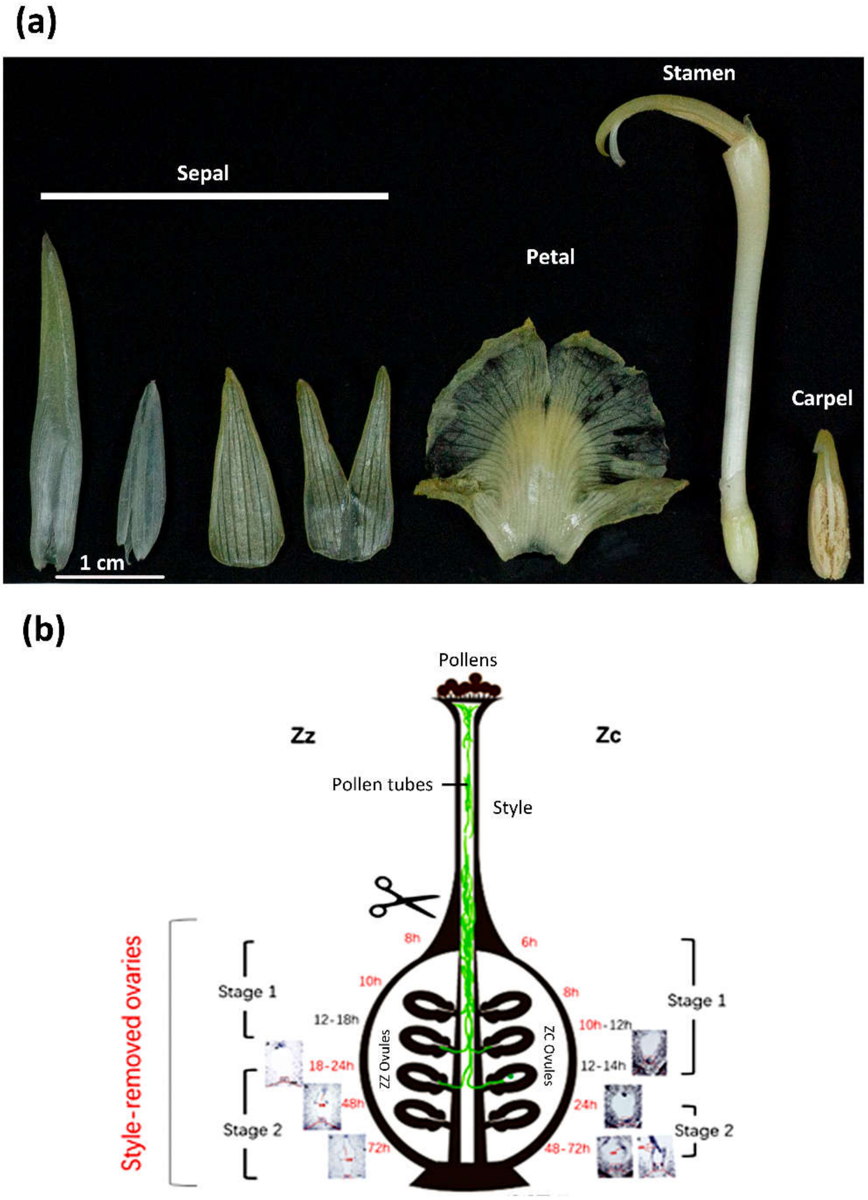

Figure S2. Megasporogenesis and female gametophyte development in ZC. Samples were collected and treated as described in Materials and Methods section. The characteristic structure are shown for each development stage. sy, synergid; ec, egg cell. Bar=20 μm. **(a).** Archesporium cell (red arrow). **(b).** The megaspore mother cell (red arrow) at early stage. **(c).** The megaspore mother cell (red arrow) at late stage. **(d).** The megaspore dyad (red arrow). **(e).** The functional megaspore (red arrow) at the chalazal end at the tetrad stage. Other three megaspores have degenerated. **(f).** The mononuclear embryo sac. The functional megaspore (red arrow) is undergoing the first run of mitosis. Condensed chromatin can be observed. **(g).** The 2-ucleate embryo sac with two nuclei (red arrow). **(h).** The 4-nucleate embryo sac. Three nuclei are shown (red arrow). **(i)-(j).** Serial sections from the same ovule. The 8-nucleate embryo sac with eight nuclei (red arrow) are shown. **(k)-(l).** Serial sections from the same ovule. The mature embryo sac with two synergids **(k)** and one egg cell **(l)** are shown.

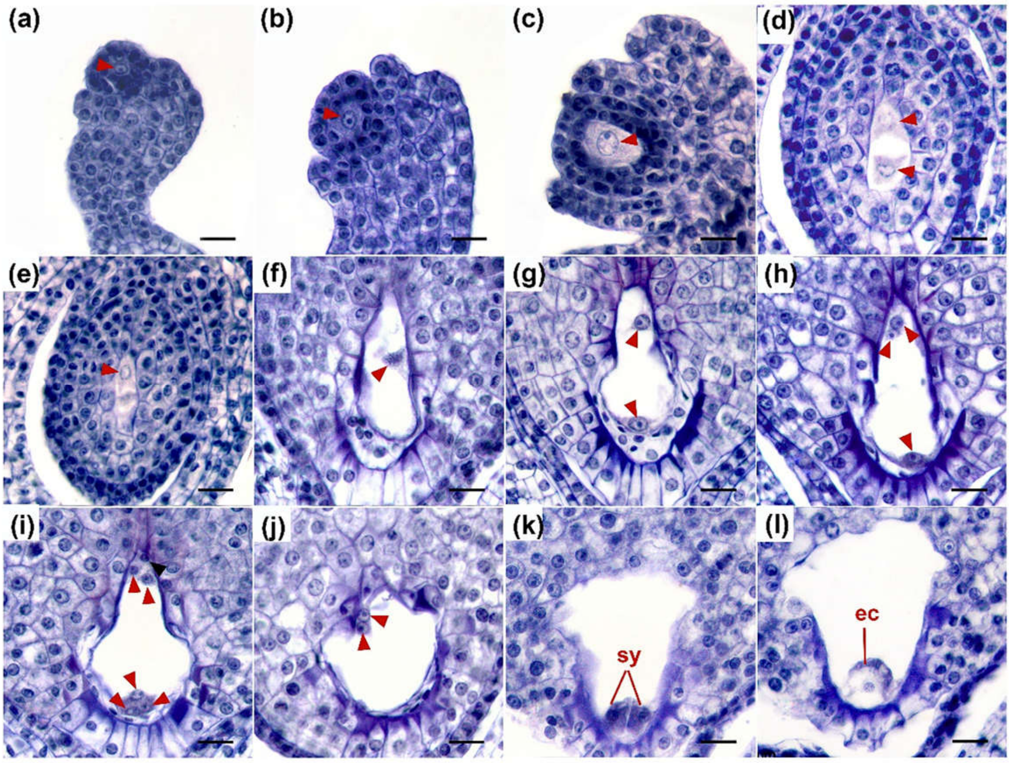

Figure S3. Megasporogenesis and female gametophyte development in ZZ. Samples were collected and treated as described in Materials and Methods section. The characteristic structure are shown for each development stage. sy, synergid; ec, egg cell. Bar=20 μm. **(a).** Archesporium cell (red arrow). **(b).** The megaspore mother cell (red arrow). **(c).** The megaspore dyad. The two daughter cells generated from cell division of the mother cell are shown by red arrowheads. **(d).** The megaspore tetrad. The functional megaspore (red arrowhead) at the chalazal end is shown. The residues of other three degenerated megaspores can be found near the end of micropyle. **(e).** The mononuclear embryo sac with one nucleus (red arrow). **(f).** The 2-nucleate embryo sac. One of the nuclei is shown (red arrow). **(g)-(h).** The 4-nucleate embryo sac. The four nuclei are shown (red arrow). **(i)-(j).** Serial sections from the same ovule. Seven nuclei (red arrow) of the 8-nucleate embryo sac are shown. **(k)-(l).** Serial sections from the same ovule containing the mature embryo sac. Two synergids **(k)** and one egg cell **(l)** are shown.

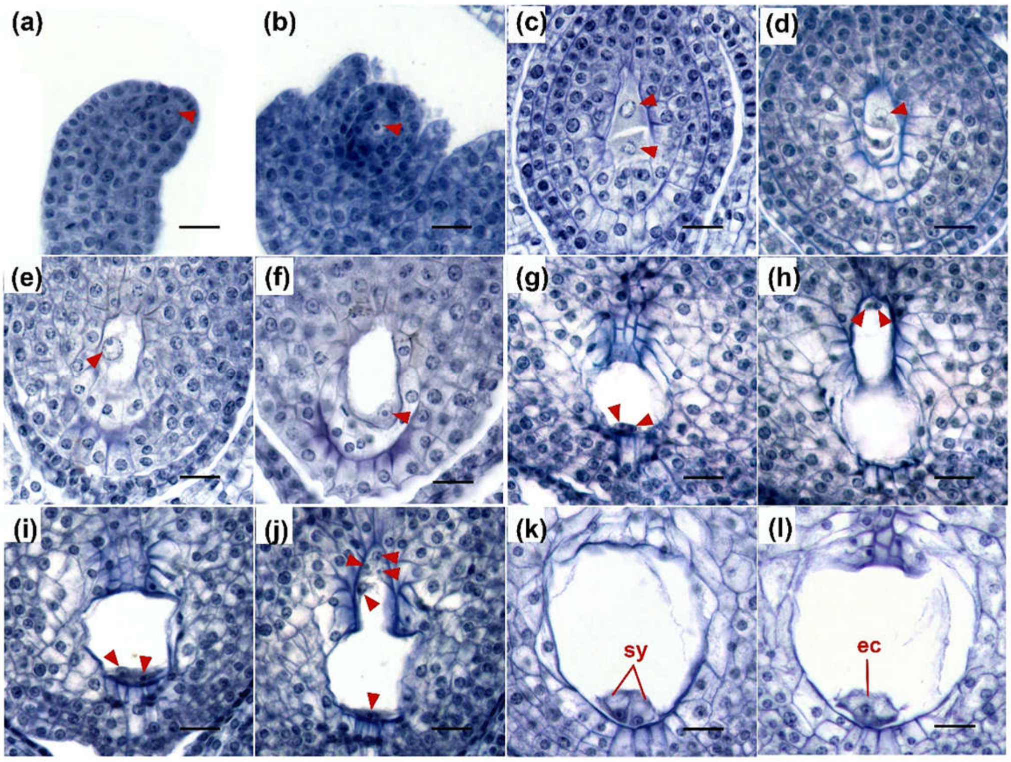

Figure S4. Microsporogenesis and male gametophyte development in ZC. (a)-(i). Microsporogenesis and the development of anther wall. **(j)-(l).** The meiosis process of the microspore mother cell. **(m)-(p).** Development of male gametophyte. Samples were collected and treated as described in Materials and Methods section. The characteristic structure are shown for each development stage. ppc, primary peripheral cell; psc, primary sporogenous cell; Ep, Epidermis; En, Endothecium; MI, Middle layer; Ta, Tapetum; vn, vegetative nucleus; gc, genetative cell. **(a).** The archesporium cell (red arrow) stage. **(b).** The archesporium is divided into a primary parietal cell (ppc) and a primary sporogenous cell (psc). **(c).** The anther wall at early stage. The indicated five layers of cells are differentiated from the secondary peripheral cells (from outside to inside: epidermis, endothecium, middle layer, and tapetum). **(d).** The sporogenous cells (red arrow) and the seven layers anther wall (from outside to inside: epidermis, endothecium, middle layer, and tapetum). **(e).** The microspore mother cells and the thickest connective (red arrow). **(f).** The tapetum undergoing degradation (red arrow). **(g).** The microspores dyads stage. The tapetum undergoing-degradation (red arrow) is shown. **(h).** The anther at 2-nucleate pollen stage. Red arrow indicates the connective. **(i).** Dehisced anther. Red arrow indicates the dehiscence area. **(j).** Microspore mother cell **(k)** Microspores dyads. **(l).** Microspores tetrad. **(m).** Mononuclear microspore in early stage **(n).** Mononuclear marginal stage. **(o).** Two-celled pollen. **(p).** Mature pollen grain (2-celled) with vegetative cells and generative cell. Bar=20µm of (a)-(d), (f),(g),(j)-(p). Bar=50µm of (e). Bar=100µm of (h) and (i).

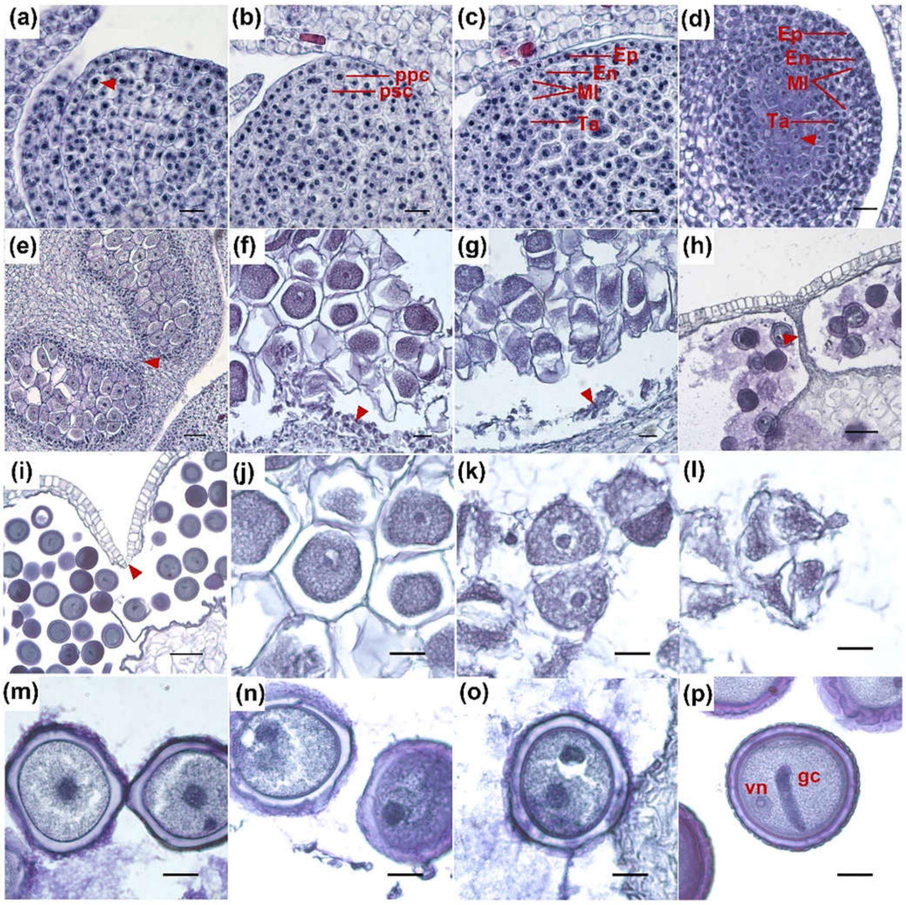

Figure S5. Microsporogenesis and male gametophyte development in ZZ. (a)-(i). Microsporogenesis and the development of anther wall. **(j)-(l).** The meiosis process of the microspore mother cell. **(m)-(p).** Development of male gametophyte. Samples were collected and treated as described in Materials and Methods section. The characteristic structure are shown for each development stage. ppc, primary peripheral cell; psc, primary sporogenous cell; Ep, Epidermis; En, Endothecium; Ml, Middle layer; Ta, Tapetum; vn, vegetative nucleus; gc, generative cell. Archesporium cell (red arrow) stage. **(a).** The archesporium presented underneath the epidermis of the pollen grain. **(b).** The archesporium is divided into a primary parietal cell and a primary sporogenous cell. **(c).** The anther wall at early stage. The indicated five layers of cells are differentiated from the secondary peripheral cells (from outside to inside: epidermis, endothecium, middle layer, and tapetum). **(d).** The sporogenous cells (red arrow) and the seven layers anther wall (from outside to inside: epidermis, endothecium, middle layer, and tapetum). **(e).** The microspore mother cells and the thickest connective (red arrow). **(f).** The tapetum undergoing degradation (red arrow). **(g).** The microspores dyads stage. The tapetum undergoing-degradation (red arrow) is shown. **(h).** The anther at 2-nucleate pollen stage. Red arrowhead indicates the pollen with 2 nuclei. The blue arrowhead indicates the degraded tapetum. **(i)**. Dehisced anther. Red arrow indicates the dehiscence area. **(j).** Microspore mother cell **(k)**. Microspores dyads. **(l)**. Microspores tetrad. **(m)**. Mononuclear microspore in early stage **(n)** Mononuclear marginal stage. **(o)**. Two-celled pollen. **(p)**. Mature pollen grain (2-celled) with vegetative cells and generative cell. Bar=20 μm for (a)-(d), (f), and (j)-(p). Bar=50 μm for (e). Bar=100 μm for (g)- (i).

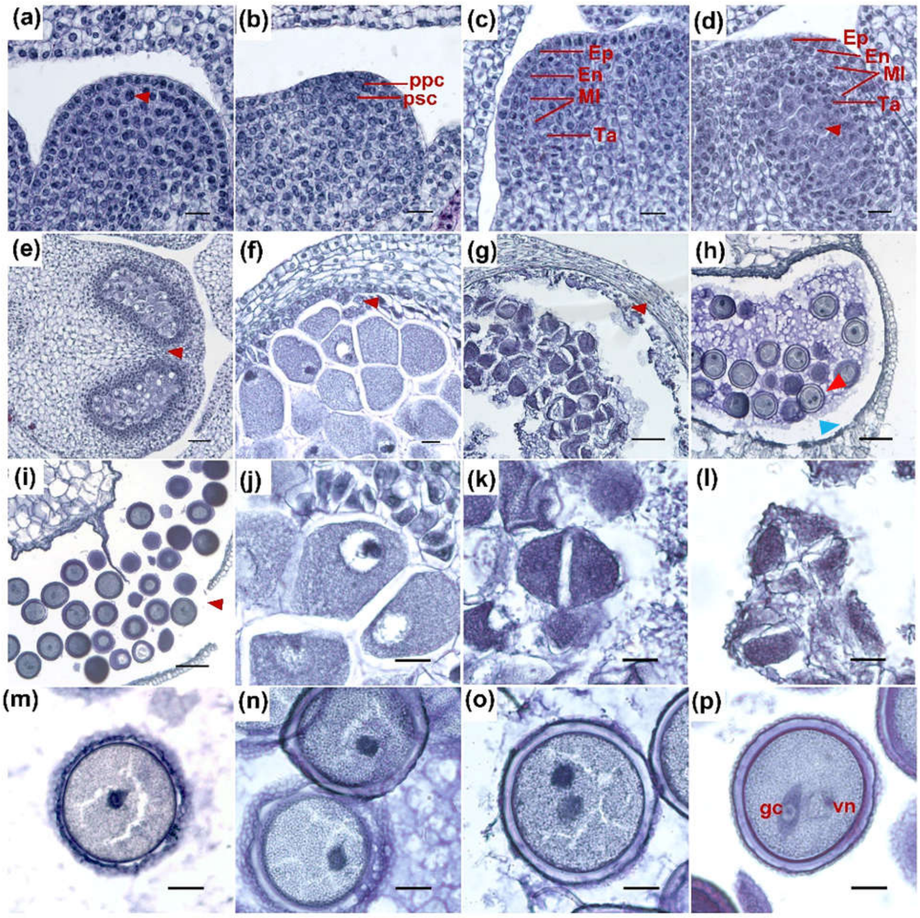

Figure S6. Viability of pollen and stigma. (a). MTT assay. Pollens and stigmas were collected at 9 AM and the viability of pollen and stigma were tested using MTT method. **(b)**. Time-course change of the viability of pollen and stigma. Pollens and stigmas were collected at different time points in a day. The viability were tested using MTT method immediately after collection. Percent of stained pollens or stigmas were calculated and shown in mean±SE of at least two replicates.

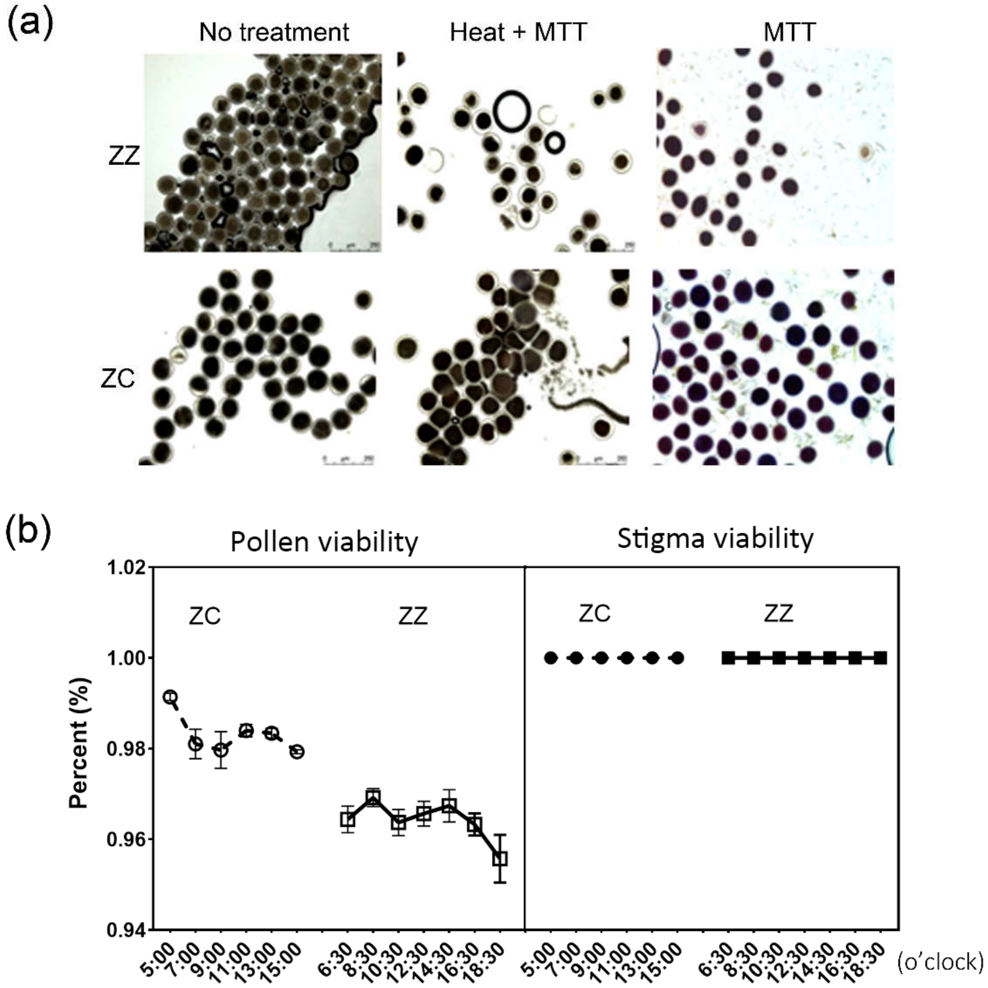

Figure S7. ZZ Pollen tubes growth inside the ovary. (A) The style of ZZ. The yellow box marks the sampling site of the ovaries used in subfigure (B-G). Sampling site for the subfigure (b-g) is marked by the red box. **(B-G)** Pollen tubes grow through the entrance of the ovary. Ovaries were sampled at (B) 10 AHP, (C) 12 HAP, (D) 14 HAP, (E) 16 HAP, (F) 18 HAP, or (G) 20 HAP. Red arrows indicate the clusters of pollen tubes. **(b-g)** Pollen tubes arriving at the ovules. The ovary samples used are the same as those in (B-G). Red arrows indicate the presence of pollen tubes at the funiculus attaching sites at (b) 10 AHP, (c) 12 HAP, (d) 14 HAP, (e) 16 HAP, (f) 18 HAP, or (g) 20 HAP. **(H-K)** Pollen tubes invading into the ovules. The ovary sample used in this experiment were collected and prepared as described in Materials and Methods section. Photographs of ovules were taken at (H) 12 h, (I) 14 h or (J-K) 16 h. The red arrow indicate a pollen tube that was going into the ovule. **(L)** The longitudinal section of a ZZ ovary. A typical picture is shown. **(M)** The longitudinal section of a ZZ ovary at 16 HAP. The invading pollen tube is indicated by red arrow. **(N-P)** Pollen tube invade into the embryo sac at 24 HAP. Three consecutive slices of the longitudinal section of a ZZ ovary were prepared. The pictures were taken for the same ovule. Red arrows indicate the path of the pollen tube.

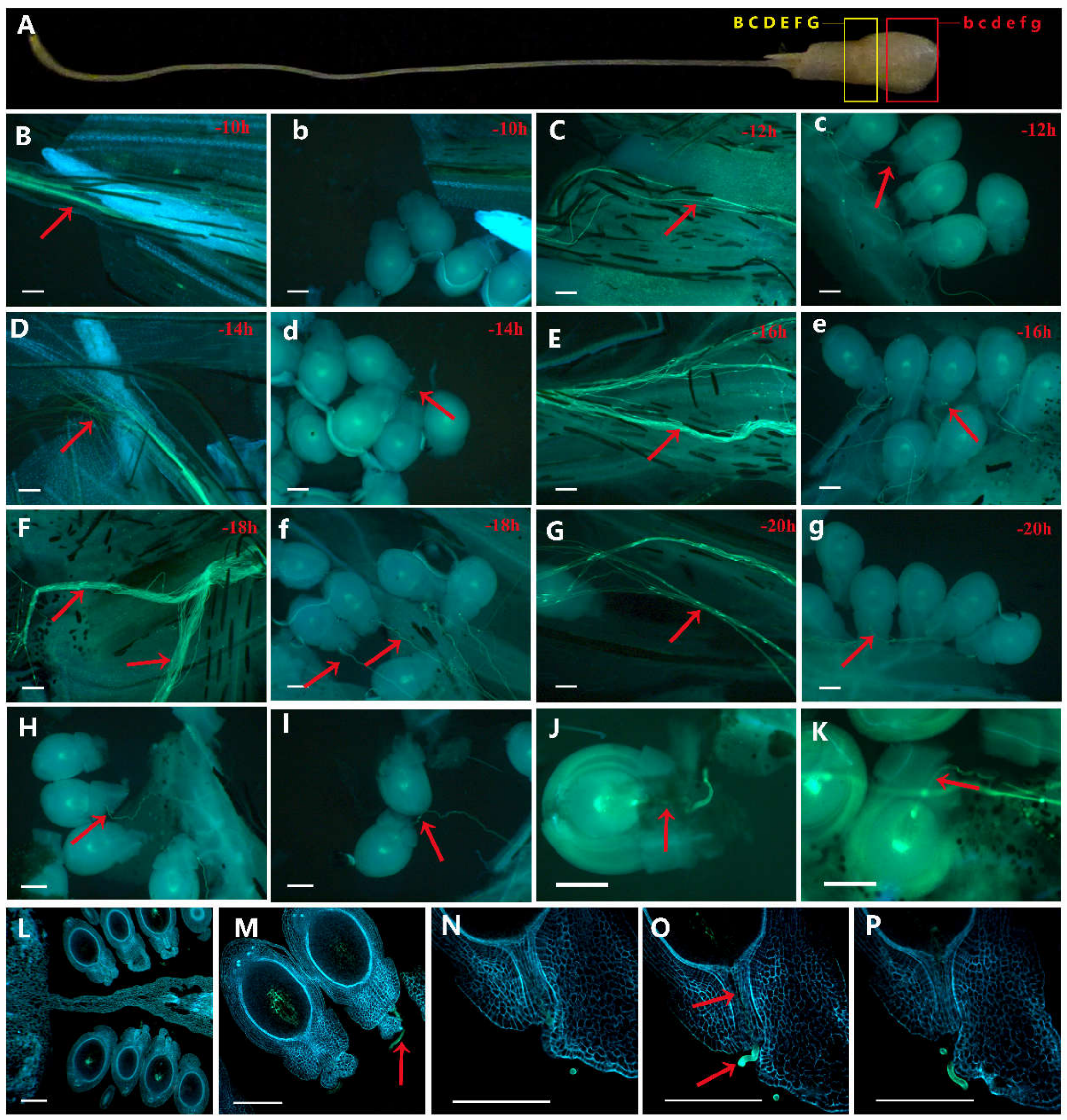

Bar=200µm.

Figure S8. De novo assembly and GO annotations for ZC and ZZ unigenes. (a) Length distribution of the generated transcripts and unigenes. **(b)** Frequency distribution of FPKM values. **(c)** ZZ and ZC unigenes are annotated based on GO annotations of *Arabidopsis* homologs and assigned to functional categories.

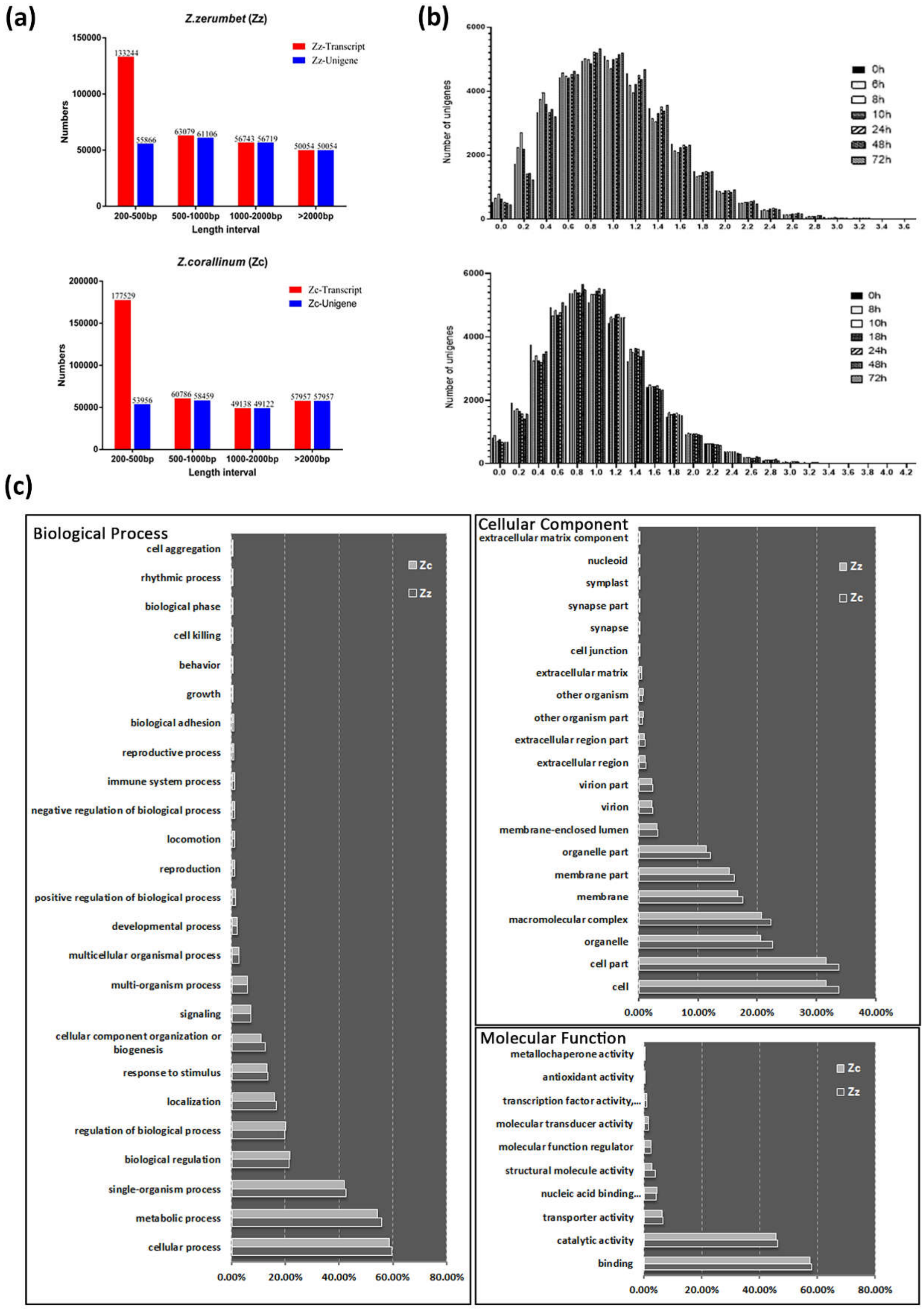

Figure S9. Real time qPCR and PCA analysis. (a) Real time qPCR verification for selected unigenes. Unigenes that are functional in reproduction in prediction were selected as the detected targets. The columns show the data from RNA-seq, while the curves indicate the expression changes detected by qRT-PCR analysis. Primers are listed in Table S4. **(b)** Principle component analysis for expression of ZZ and ZC unigenes.

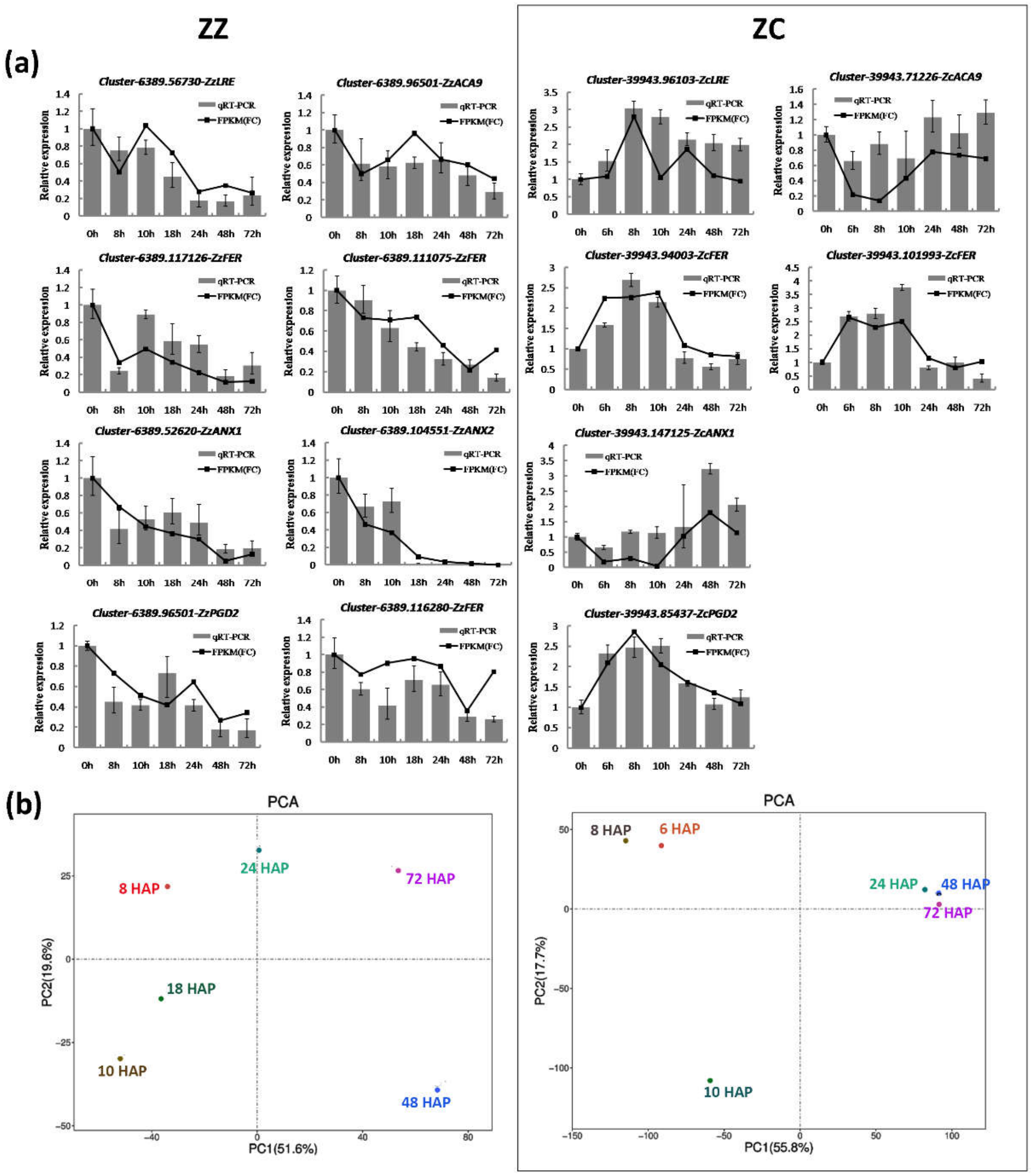

Figure S10. Twenty time-course profiles identified from ZZ and ZC SBS genes. The numbers of genes involved in each profile are indicated at the left-down for ZZ (left) and ZC (right), respectively. The significant overrepresented profiles are highlighted by * for ZZ and by ** for ZC.

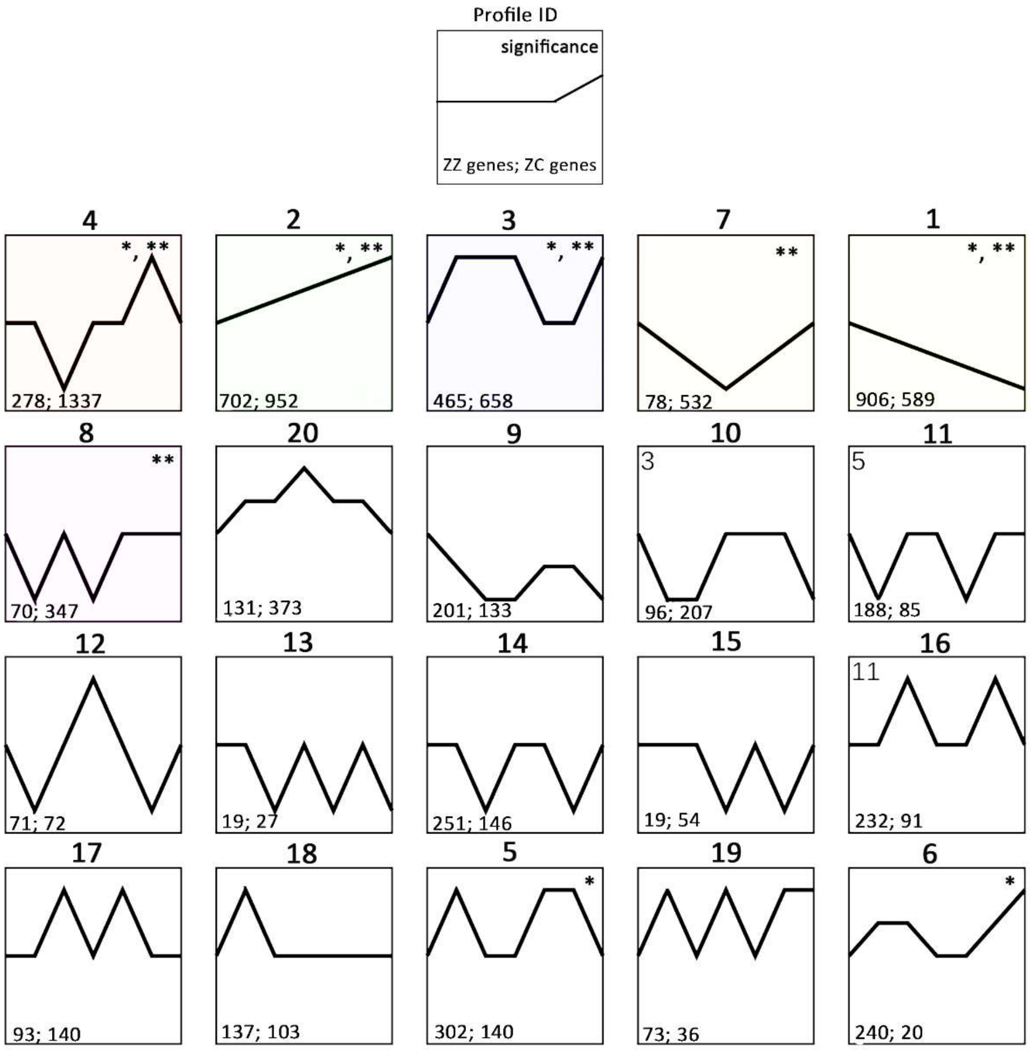

Figure S11. Phylogenetic analysis for homologs of FER and LRE proteins. (a) Unigenes containing full-length ORF are retrieved and the deduced amino acid sequences are used in phylogenetic analysis together with *Arabidopsis* homologs. ClustalW method was adopted to perform alignment using MegAlign in DNAStar package under default setting. **(b)** Alignment of ANX proteins of ZZ, ZC and *Arabidopsis.*

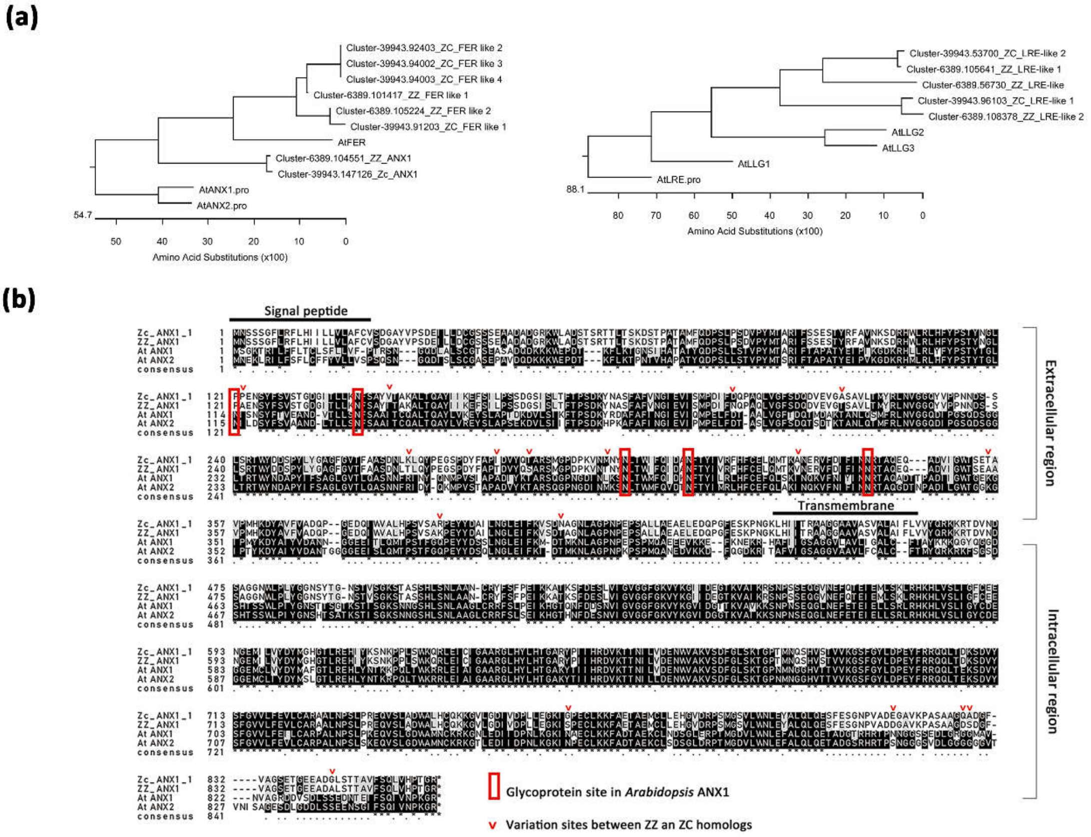

Figure S12. Alignment of the predicted RALF19 and RALF34 peptides from ZZ, ZC and Arabidopsis thaliana. Red "^" indicates the variation site between ZZ and ZC.

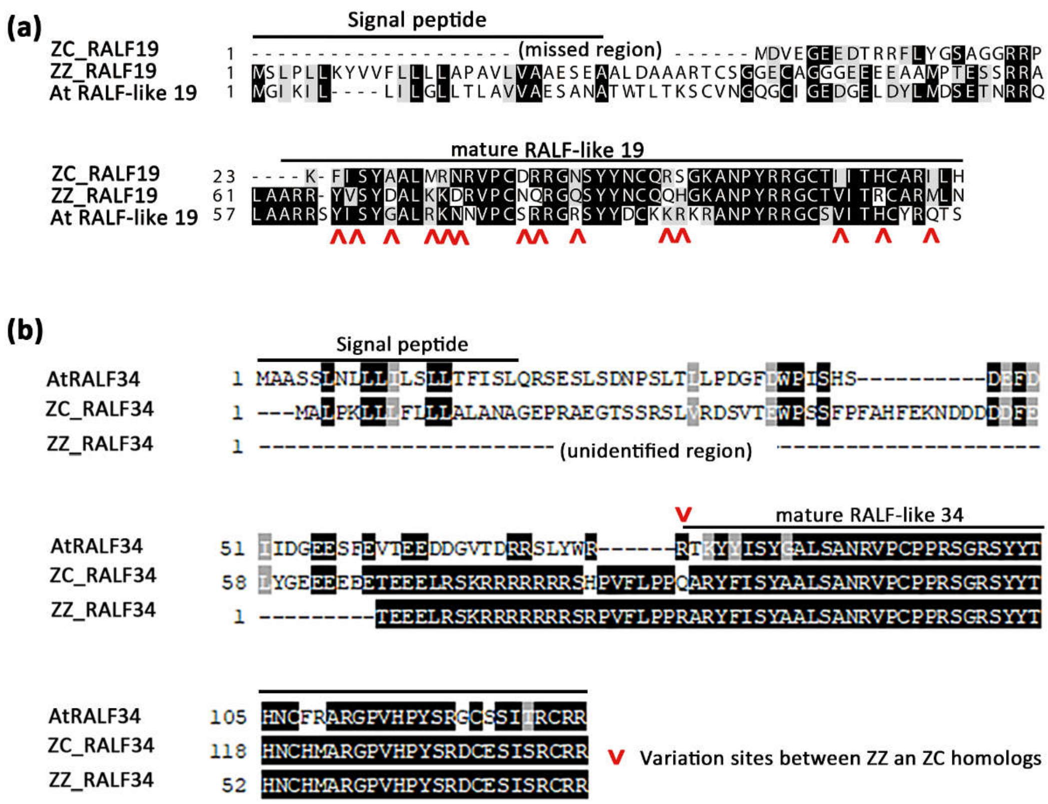

Figure S13. ZZ and ZC Orthologs for MPK and PRK proteins. (a) ML tree of MPK proteins. **(b)** ML tree of PRK proteins. **(c)** Prediction of the signal peptide and the transmembrane region of the PRK proteins.

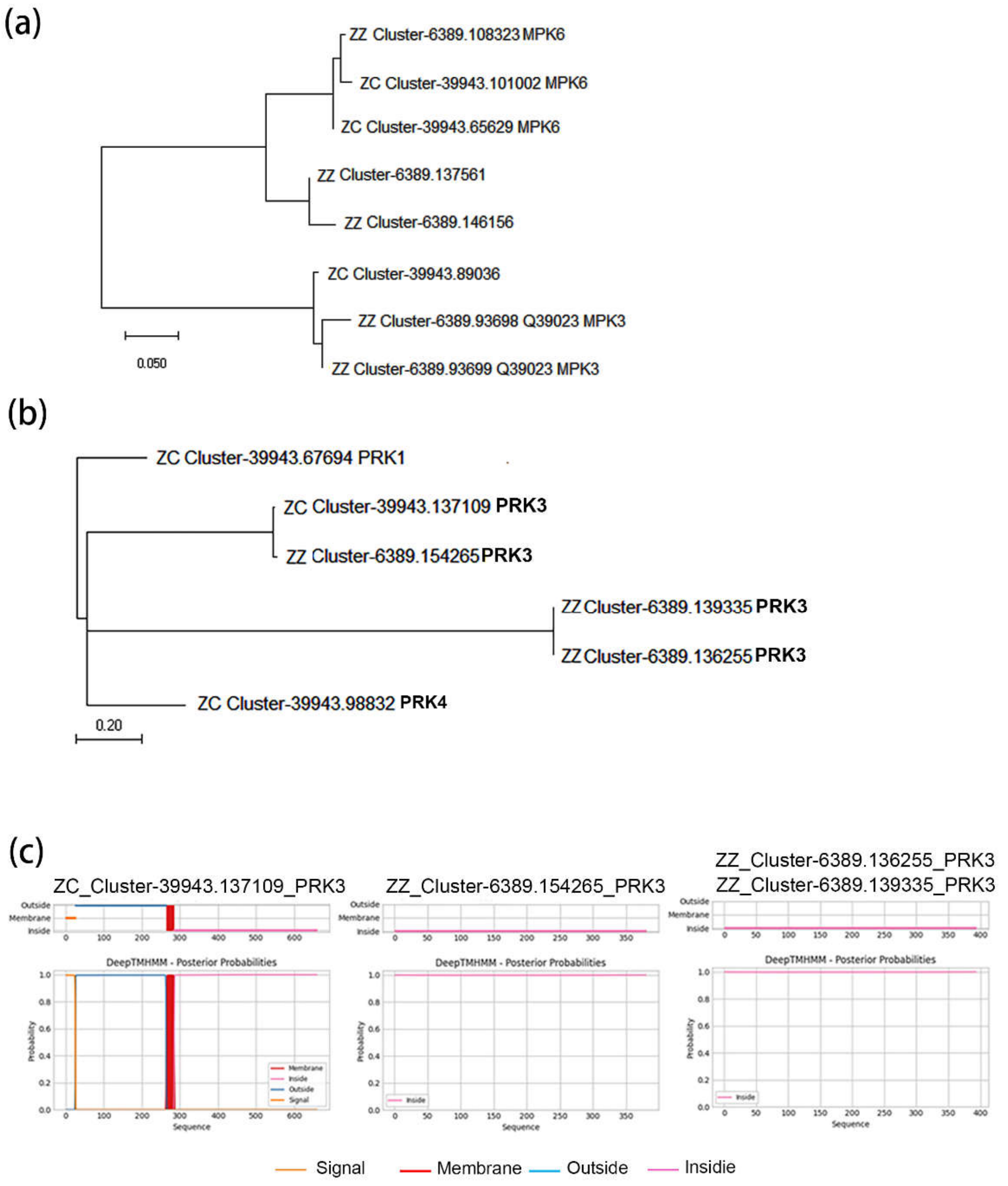

Supplement: Supplementary file 1 [file DataSheet_1.pdf]
